# Supplementary material for: Detailed Analysis of the Genetic and Epigenetic Signatures of iPSC-Derived Mesodiencephalic Dopaminergic Neurons
Source: Stem Cell Reports. 2014 Apr 3;2(4):520–33. doi: 10.1016/j.stemcr.2014.03.001 (PMC3986662; doi:10.1016/j.stemcr.2014.03.001)
Supplement: Document S1. Supplemental Experimental Procedures, Figures S1–S5, and Table S1 [file mmc1.pdf]

Stem Cell Reports, Volume *2*

Supplemental Information

Detailed Analysis of the Genetic and Epigenetic Signatures of iPSC-Derived Mesodiencephalic Dopaminergic Neurons

Reinhard Roessler, Sebastien A. Smallwood, Jesse V. Veenvliet, Petros Pechlivanoglou, Su-Ping Peng, Koushik Chakrabarty, Marian J.A. Groot-Koerkamp, R. Jeroen Pasterkamp, Evelyn Wesseling, Gavin Kelsey, Erik Boddeke, Marten P. Smidt, and Sjef Copray

Inventory of Supplemental Information

Supplemental Experimental Procedures:

FAC sorting

Electrophysiology

Rat rotation model

Immunocytochemistry

Western Blot

Direct bisulfite sequencing

Quantitative PCR (qPCR)

Quantitative dopamine determination by LC-MS/MS

Reverse transcriptase PCR

Gene ontology analysis

Supplemental References

Supplemental Table: 1

Table S1: Primer sequences used for RT- and quantitative RT-PCR

Supplemental Figures: 4

Figure S1, related to Figure 1: Generation of iPSCs from *Pitx3*$^{Gfp/+}$ MEFs.

Figure S2, related to Figure 1: PITX3-GFP sorted DA neurons function *in vivo*

Figure S3, related to Figure 2: Extended expression profiling of mdDA signature.

Figure S4, related to Figure 3: DNA methylation profile for key DA transcription factors.

Figure S5, related to Figure 4: Analysis of gene expression profiles vs. DNA methylation patterns in mdDa neurons and iPSC-DA neurons respectively.

Supplemental Experimental Procedures

FAC sorting

Freshly dissected PITX3-GFP positive ventral midbrains from several developmental stages, undifferentiated and differentiated iPSC-derived cells were dissociated using the Papain dissociation Kit (Worthington Biochem, Products, LK003150). Dissociated cells were collected in colourless DMEM (Gibco) with 20 ng/ml BDNF, 20 ng/ml GDNF, 0.2 mM ascorbic acid, 1 ng/ml TGFb3 and 0.1 mM dbcAMP, and sorted on a MoFlo-XDP or MoFlo-Astrios sorter with a 100 um nozzle at a pressure of 15-17 psi based on GFP expression. Cells were collected in N2 medium with BDNF, ascorbic acid, GDNF, TGFb3 and dbcAMP or cells were collected in PBS, pelleted and used for RNA and DNA isolation.

Electrophysiology

For patch clamp experiments, FAC-sorted iPSC-derived *Pitx3*$^{Gfp/+}$ cells were spun down at 800 rcf for 10 min, resuspended and plated on mouse astrocyte feeders. Electrophysiological recordings were performed on *Pitx3*$^{Gfp/+}$ neurons one week after plating. A standard whole-cell patch clamp technique was used to measure currents at room temperature (22–24°C) using Axopatch 200B amplifier, Digidata 1320A, and pClamp version 8.2 software (Axon Instruments, Foster City, CA). Extracellular bath solution contained (in mmol/L): 140 NaCl, 4 KCl, 5 glucose, 2 $CaCl_2$, 1 $MgCl_2$, and 10 HEPES, pH adjusted to 7.4 with NaOH. Pipette solution contained (in mmol/L) 140 KCl, 10 EGTA, 1 $CaCl_2$, 1 $MgCl_2$ and 10 HEPES,2 Na_2ATP, pH adjusted to 7.2 with KOH and had an osmolality of 290-300 mOsm. Microelectrodes were made of GC120F- 10 borosilicate glass (Harvard Apparatus) and pulled on a P-87 puller (Sutter Instruments, Novato, CA) having a final resistance of 4–5 MΩ. Series resistance was compensated by 80%–85%. Currents were filtered at 5 kHz and digitized at 10 kHz. Data were analyzed using Clampfit (Axon Instruments) and Excel (Microsoft). In the current clamp mode cell membrane potential was held at −50 mV by injecting a steady holding current. Cells were activated with a 500 ms pulse of depolarizing current.

Rat rotation model

To test functionality of iPSC-derived DA neurons, we have used the well-established rat rotation model (Ungerstedt, 1971). Adult female Sprague Dawley (Harlan) rats (180–230g) were housed under standard conditions with free access to food and water. All animal experiments were carried out

according to the Dutch Regulations for Animal Welfare. Protocols were approved by the Institutional Animal Care and Use Committee of the University of Groningen. Rats were anaesthetized with ketamine (90mg/kg) and xylazine (4mg/kg). Unilateral retrograde destruction of dopaminergic neurons in the substantia nigra was induced by 2 stereotaxic injections of 2.5 µl 6-OHDA (3 mg/ml in 0.2% ascorbic acid and 0.9% saline, Sigma) in the medial forebrain bundle of the nigro-striatal pathway (stereotaxic coordinates 1: AP -4.0, ML −0.8 and V −8.0; tooth bar set at +3.4. and coordinates 2: AP -4.4, ML −1.2 and V −7.8; tooth bar set at −2.4). Unilateral destruction of the nigro-striatal pathway was established by recording of d-amphetamine (5mg/kg)-induced rotation behavior after 3, 6 and 9 weeks. Rats were selected for transplantation if the number of rotations exceeded 4 per min at 6–8 weeks post injection. Under ketamine/metedomedine anesthesia, 2-3*10^4 cells were stereotactically transplanted into the striatum (coordinates: AP +0.9mm, ML −2.6mm and V −4.0mm; tooth bar set at 0.0); control rats received a similar injection with mouse embryonic fibroblasts. Daily intraperitoneal injections of cyclosporine 15mg/kg (UMCG pharmacy) were given, starting 24 h before cell grafting (double dosage), and continued until the rats were sacrificed and perfusion fixated at 9 weeks after grafting. Under ketamine/metedomedine anesthesia, rats were transcardially perfused with 4% paraformaldehyde (PFA) in PBS. Brains were explanted, post-fixed in 4% PFA for 24 hours and soaked in a 20 % sucrose solution for 1 day. Brains were sectioned (14µm sections) on a cryostat after embedding in O.C.T. compound (Sakura Finetek, Torrance, USA).

Immunocytochemistry

Cells were fixed in 4% paraformaldehyde for 15 min and blocked with 5% normal goat serum, 2% fetal calf serum in 0,1% Triton X in PBS. To characterize iPSC colonies, primary antibodies for Nanog (1:500, Abcam, ab80892), Oct3/4 (1:500, sc-5279, Santa Cruz), Klf4 (1:500, Abcam, ab72543), Sox2 (1:500, Abcam, ab15830), UTF1 (1:1000, mUTF1 custom made rabbit polyclonal, Eurogentec, (Van den Boom et al., 2007)) and alkaline phosphatase (1:500, Abcam, ab65834) were used. To characterize iPSC-derived neurons, primary antibodies for Map2 (1:500, Millipore, AB5622), TH (1:1000, Millipore, ab152) and GFP (1:500, Abcam, ab290) were used. To visualize primary antibodies, fluorescently labeled secondary anti-mouse and anti-rabbit antibodies were applied. Nuclear staining was performed using Hoechst.

Western Blot

SDS-PAGE gel electrophoresis and Western Blot analysis were performed to detect Nanog, Oct3/4, Sox2 and UTF1. The following antibodies were used: rabbit anti-Nanog antibody (1:1000, ab80892, Abcam, Cambridge, UK), mouse Oct3/4 antibody (1:500, sc-5279, Santa Cruz), rabbit anti-Sox2 (1:4000, ab15830, Abcam, Cambridge, UK), rabbit UTF1 (1:2000) and mouse anti-β-Actin (ab6276, Abcam, Cambridge, UK) at 1:10000 dilution. The housekeeping gene β-Actin has been used as loading control. Primary antibodies were detected using fluorescently labeled secondary antibody: donkey anti-mouse (IRDye® 680, LI-COR, Biosciences) and donkey anti-rabbit (IRDye® 800CW, LI-COR, Biosciences) according to manufactures instructions.

Direct bisulfite sequencing

Genomic DNA from Pitx3$^{gfp/+}$ iPSCs was purified by proteinase K digestion followed by phenol-chloroform extraction. Sodium bisulfite conversion was carried out with the Zymo EZ DNA methylation Kit. PCRs were performed for Oct4 (FW: 5'-GTGTAGTGTTAATAGGTTTT-GTGG-3'; BW: 5' TATTTTTTAATTTTAA-TTTTTA3'), Nanog (FW: 5’GGATTTTGTAGG-TGGGATTAAT 3’; BW: 5’ TAAACAACAACCAAAAAACTCAA 3’). Sequence analysis of converted fragments was done using Quma Quantification tool for methylation analysis (quma.cdb.riken.jp).

Quantitative PCR (qPCR)

Relative gene expression levels were determined by qPCR real-time pcr (Lightcycler) using the QuantiTect™ SYBR® Green PCR (QIAGEN) LightCycler® kit (Roche, Idaho Technologies) according to the manufacturer’s instructions. For each reaction 0.1ng total RNA from FAC-sorted neurons was used as input. Relative expression levels were normalized to housekeeping gene *18s*. Primer sequences see Table 1.

Quantitative dopamine determination by LC-MS/MS

Dopamine production was determined as described earlier (Van de Merbel et al., 2011). Instead of plasma we analyzed 100µl of supernatant or cell homogenate from terminally DA differentiated iPSCs or undifferentiated iPSCs (3 technical replicates each) for the derivation step.

Reverse transcriptase PCR

Briefly, RNA was isolated using the RNA-easy kit (Qiagen). To characterize endogenous expression of pluripotency markers in *Pitx3*$^{Gfp/+}$ iPSC clones 3 and 5, the following primer genes were analyzed: Nanog, Oct4, Sox2, Klf4 and cMyc (primer sequences as published by Takahashi and Yamanaka, 2006, see Table 1). Expression levels were compared between two iPSC clones, ES cell line IB10 and mouse embryonic fibroblasts (C57BL/6).

Gene ontology analysis

GO analysis on transcriptome data was performed using the BiNGO 2.44 plug-in (Cytoscape 2.8.2; Hypergeometric test; FDR correction) using genes with an at least 2-fold up– or down-regulation in iPSC-derived DA neurons as compared to their primary counterpart at any developmental time point as input, and all annotated genes on the micro-array as reference. GO analysis for the RRBS data set (see below for RRBS) was performed using online functional annotation tool DAVID (Huang et al., 2009). The list of genes analyzed with DAVID was generated based on the entire RRBS data set. To select most different genes CGI methylation reads were sorted and plotted after logit transformation (see Figure S5). Histogram plots were created with the statistical software R v.2.15.2 [R Core Team (2012).

Supplemental References

Hargus, G., Cooper, O., Deleidi, M., Levy, A., Lee, K., Marlow, E., Yow, A., Soldner, F., Hockemeyer, D., Hallett, P.J., Osborn, T., Jaenisch, R., Isacson, O. (2010). Differentiated Parkinson patient-derived induced pluripotent stem cells grow in the adult rodent brain and reduce motor asymmetry in Parkinsonian rats. Proc. Natl. Acad. Sci. USA *107*, 15921–6.

Ungerstedt, U. (1971). Postsynaptic supersensitivity after 6-hydroxy-dopamine induced degeneration of the nigro-striatal dopamine system. Acta Physiol. Scand. Suppl. *367*, 69–93.

Van de Merbel, N.C., Hendriks, G., Imbos, R., Tuunainen, J., Rouru, J., Nikkanen, H. (2011). Quantitative determination of free and total dopamine in human plasma by LC-MS/MS: the importance of sample preparation. Bioanalysis *3*, 1949–61.

Van den Boom, V., Kooistra, S.M., Boesjes, M., Geverts, B., Houtsmuller, A.B., Monzen, K., Komuro, I., Essers, J., Drenth-Diephuis, L.J., Eggen, B. (2007). UTF1 is a chromatin-associated protein involved in ES cell differentiation. J. Cell Biol. *178*, 913–24.

Table S1: Primer sequences used for RT- and quantitative RT-PCR

Gene	Forward primer (5' to 3')	Reverse primer (5' to 3')
RT-PCR		
Nanog	CAGGTGTTTGAGGGTAGCTC	CGGTTCATCATGGTACAGTC
Oct4	TCTTTCCACCAGGCCCCCGGCTC	TGCGGGCGGACATGGGGAGATCC
Sox2	TAGAGCTAGACTCCGGGCGATGA	TTGCCTTAAACAAGACCACGAAA
cMyc	TGACCTAACTCGAGGAGGAGCTGG AATC	AAGTTTGAGGCAGTTAAAATTATGG CTGAAGC
Klf4	GCGAACTCACACAGGCGAGAAACC	TCGCTTCCTCTTCCTCCGACACA
GAPDH	CATCAAGAAGGTGGTGAAGC	ACCACCCTGTTGCTGTAG
qRT		
18s	AAACGGCTACCACATCCAAG	CCTCCAATGGATCCTCGTTA
Desmin	GCGGCTAAGAACATCTCTGAGG	ATCTCGCAGGTGTAGGACTGGA
Dlk1	TGGCTGTGTCAATGGAGTCTGC	CCACGCAAGTTCCATTGTTGGC
En1	GGTCTACTGCACACGCTATTCG	AACTCCGCCTTGAGTCTCTGCA
En2	GGTCTACTGCACGCGCTATTCT	AAACTCAGCCTTGAGCCTCTGG
Nurr1	CCGCCGAAATCGTTGTCAGTAC	TTCGGCTTCGAGGGTAAACGAC
Pitx3	CTTCCAGAGGAATCGCTACCCT	CTGCGAAGCCACCTTTGCACAG
Sox9	CACACGTCAAGCGACCCATGAA	TCTTCTCGCTCTCGTTCAGCAG

Figure S1, related to Figure 1

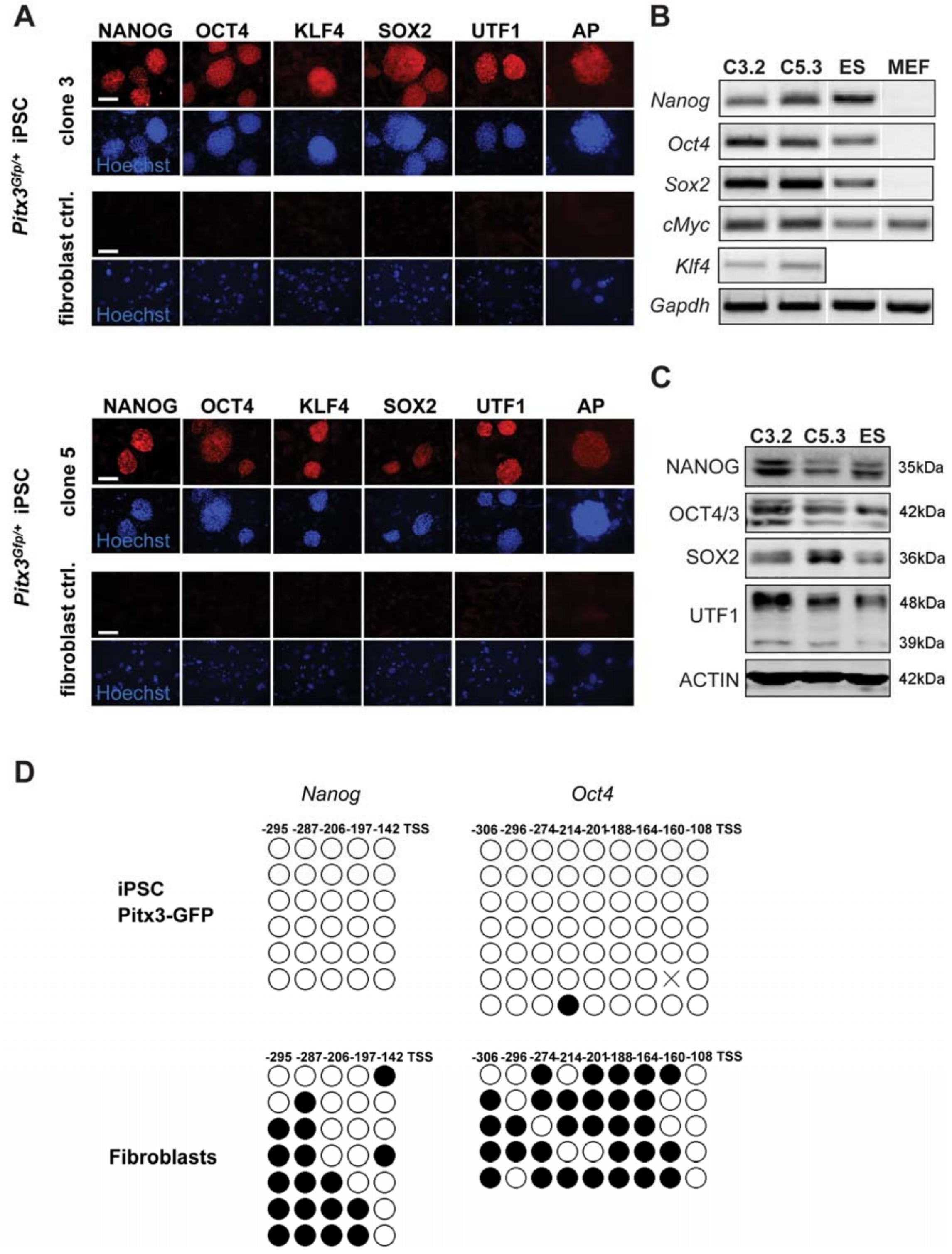

Figure S1, related to Figure 1: Generation of iPSCs from *Pitx3*$^{Gfp/+}$ MEFs. (A) Characterization of iPSC clone 3 and 5 using immune reactivity for selected pluripotency markers (blue=Hoechst nuclear staining; red=immunostaining for the indicated (transcription) factor) Scale bars for all: 100µm. **(B)** Endogenous expression of pluripotency markers comparing iPSC clones 3 and 5 with embryonic stem cells (ES) and MEFs. **(C)** Protein expression of selected pluripotency markers comparing iPSC clones

3 and 5 with ES cells. **(D)** Bisulfite sequencing of promoter regions of Nanog and Oct4 comparing *Pitx3*$^{Gfp/+}$ iPSCs with *Pitx3*$^{Gfp/+}$ MEFs. Open circles represent unmethylated CpG dinucleotides, whereas closed circles indicate methylated CpG dinucleotides.

Figure S2, related to Figure 1

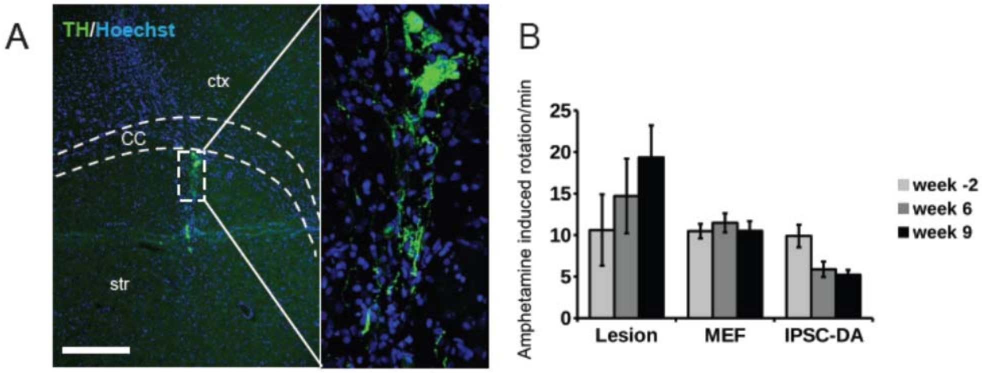

Figure S2, related to Figure 1: PITX3-GFP sorted DA neurons function *in vivo* (A) Immunohistochemistry of a section of the upper part of the rat striatum showing some of the grafted TH-positive iPSC-derived mdDA neurons with their extensive outgrowth. Scale bar = 1mm; CC=corpus callosum; str=striatum; ctx=cortex. (B) Amphetamine-induced rotation in purified *Pitx3*$^{Gfp/+}$ neuron grafted animals (n=7) vs. controls (only lesioned, n=2 and grafted MEFs, n=3; error bar = standard deviation around mean).

Figure S3, related to Figure 2

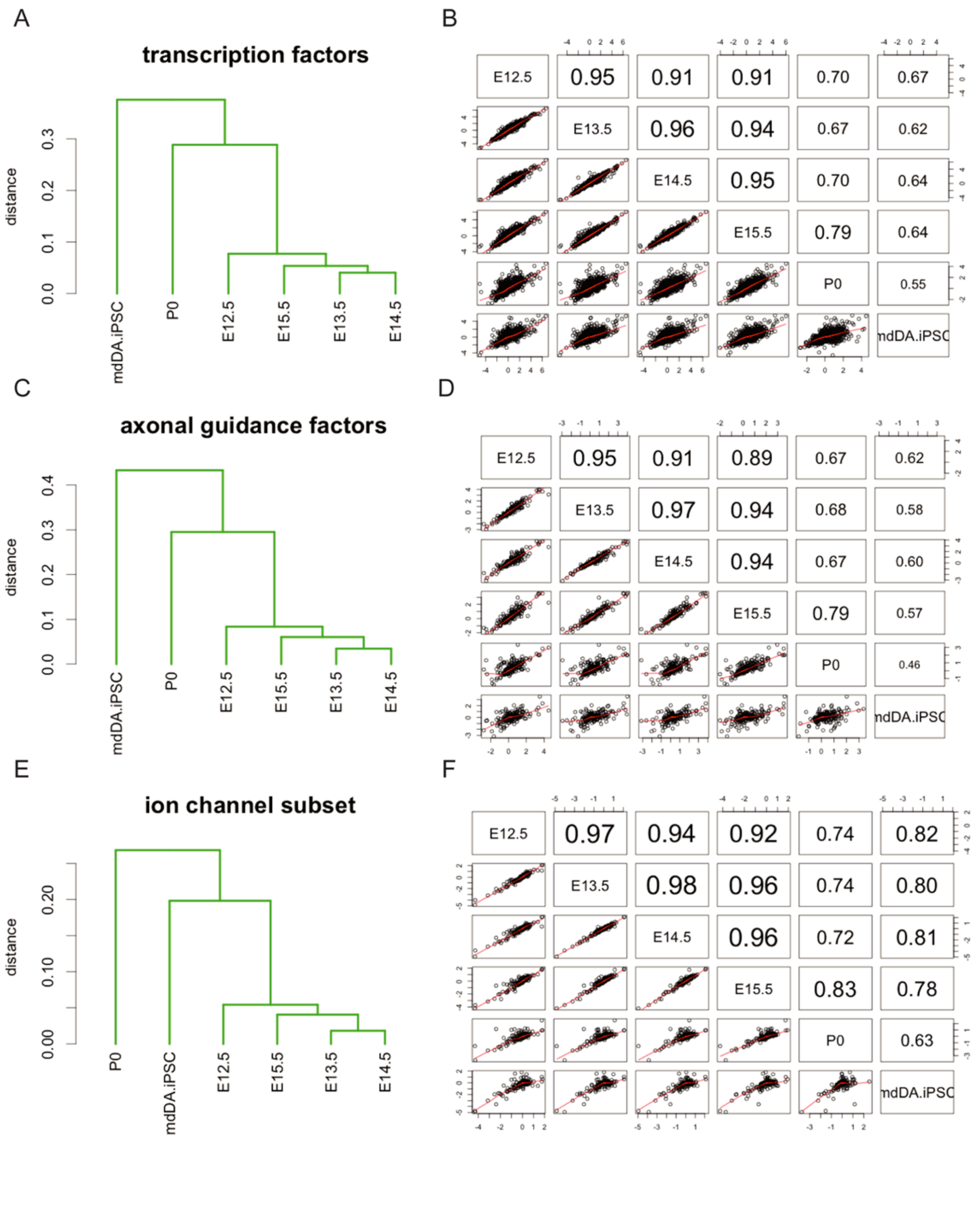

Figure S3, related to Figure 2: Extended expression profiling of mdDA signature. (A) Hierarchical clustering of DA-specific transcription factors. (B) Comprehensive analysis of expression correlation for DA-specific transcription factors. (C) Hierarchical clustering of axonal guidance factors. (D) Comprehensive analysis of expression correlation for axonal guidance factors. (E) Hierarchical clustering of ion channel subset. (F) Comprehensive analysis of expression correlation for ion channel subset.

Figure S4, related to Figure 3

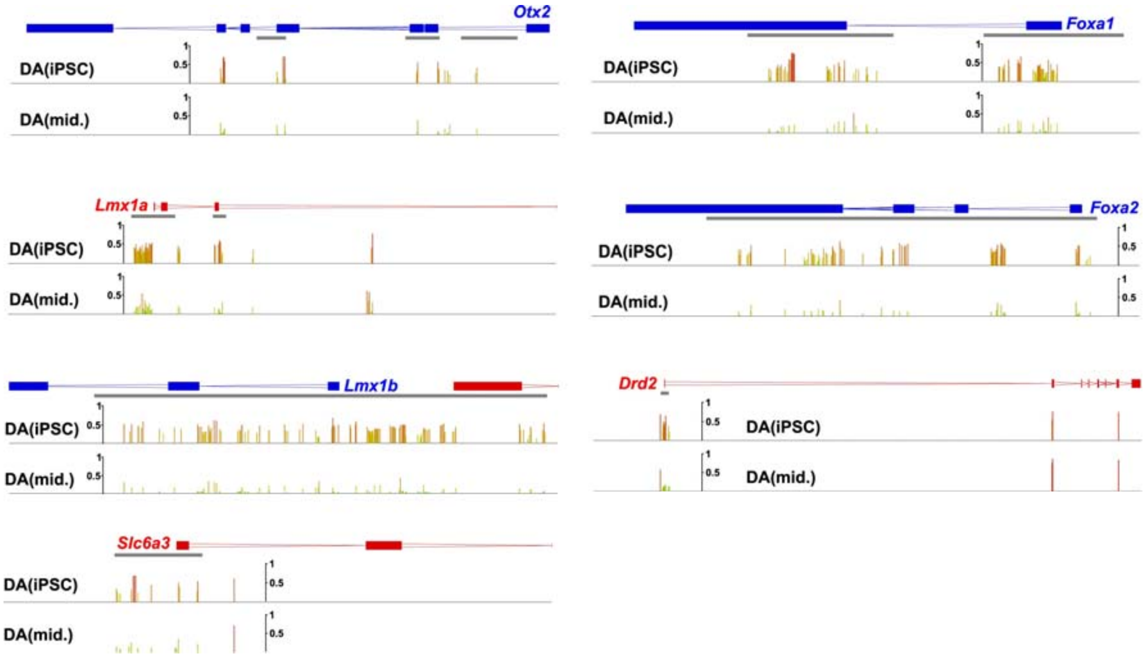

Figure S4, related to Figure 3: DNA methylation profile for key DA transcription factors. DNA methylation profile of the complete gene bodies highlighting identified CGIs for *Otx2, Lmx1a, Lmx1b*, *FoxA1/2, Slc6a3* (aka Vmat2) and *Drd2*. Most linage-specific differences can be seen in putative promoter areas. Color and arrows provide information regarding gene orientation (red = + strand; blue = - strand). Methylation across GCIs is represented in ‰.

Figure S5, related to Figure 4

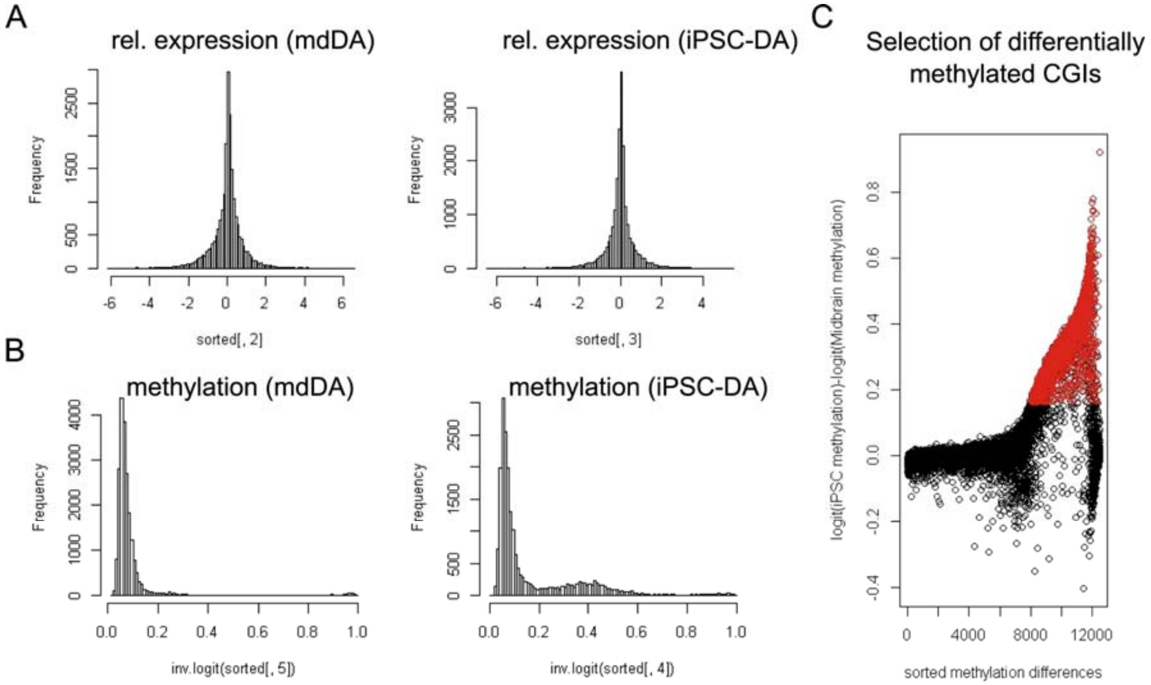

Figure S5, related to Figure 4: Analysis of gene expression profiles vs. DNA methylation patterns in mdDA neurons and iPSC-DA neurons respectively. (A) Relative global expression levels in mdDA- and iPSC-DA neurons. **(B)** Distribution of global DNA Methylation in mdDA and iPSC-DA neurons. **(C)** Identification and selection of differentially methylated CGIs in iPSC-DA neurons compared to mdDA neurons. CGIs labeled in red were selected for GO term analysis (see Figure 5).
