# Supplementary material for: Detailed Analysis of the Genetic and Epigenetic Signatures of iPSC-Derived Mesodiencephalic Dopaminergic Neurons
Source: Stem Cell Reports. 2014 Apr 3;2(4):520–33. doi: 10.1016/j.stemcr.2014.03.001 (PMC3986662; doi:10.1016/j.stemcr.2014.03.001)
Supplement: Document S2. Article plus Supplemental Information [file mmc2.pdf]

Stem Cell Reports
Article
OPEN ACCESS

# Detailed Analysis of the Genetic and Epigenetic Signatures of iPSC-Derived Mesodiencephalic Dopaminergic Neurons

Reinhard Roessler,[1,8] Sebastien A. Smallwood,[2,7] Jesse V. Veenvliet,[3,7] Petros Pechlivanoglou,[4,9] Su-Ping Peng,[1] Koushik Chakrabarty,[6] Marian J.A. Groot-Koerkamp,[5] R. Jeroen Pasterkamp,[6] Evelyn Wesseling,[1] Gavin Kelsey,[2] Erik Boddeke,[1] Marten P. Smidt,[3] and Sjef Copray[1,*]
[1]Department of Neuroscience, Section Medical Physiology, University Medical Center Groningen, 9713AV Groningen, the Netherlands
[2]Epigenetics Programme, The Babraham Institute, Cambridge CB22 3AT, UK
[3]Center for Neuroscience, Swammerdam Institute for Life Science, Science Park Amsterdam, 1098XH Amsterdam, the Netherlands
[4]Unit of Pharmacoepidemiology and Pharmacoeconomics, Department of Pharmacy, University of Groningen, 9713AV Groningen, the Netherlands
[5]Molecular Cancer Research, University Medical Center Utrecht, Universiteitsweg 100, 3584 CG Utrecht, the Netherlands
[6]Department of Neuroscience and Pharmacology, Rudolf Magnus Institute of Neuroscience, University Medical Center Utrecht, Universiteitsweg 100, 3584 CG Utrecht, the Netherlands
[7]These authors contributed equally to this work
[8]Present address: Whitehead Institute for Biomedical Research, 9 Cambridge Center, Cambridge, MA 02142, USA
[9]Present address: Toronto Health Economics and Technology Assessment (THETA), University of Toronto, Toronto ON M5S 3M2, Canada
*Correspondence: j.c.v.m.copray@umcg.nl
http://dx.doi.org/10.1016/j.stemcr.2014.03.001
This is an open access article under the CC BY license (http://creativecommons.org/licenses/by/3.0/).

## SUMMARY

Induced pluripotent stem cells (iPSCs) hold great promise for in vitro generation of disease-relevant cell types, such as mesodiencephalic dopaminergic (mdDA) neurons involved in Parkinson's disease. Although iPSC-derived midbrain DA neurons have been generated, detailed genetic and epigenetic characterizations of such neurons are lacking. The goal of this study was to examine the authenticity of iPSC-derived DA neurons obtained by established protocols. We FACS purified mdDA (*Pitx3*$^{Gfp/+}$) neurons derived from mouse iPSCs and primary mdDA (*Pitx3*$^{Gfp/+}$) neurons to analyze and compare their genetic and epigenetic features. Although iPSC-derived DA neurons largely adopted characteristics of their in vivo counterparts, relevant deviations in global gene expression and DNA methylation were found. Hypermethylated genes, mainly involved in neurodevelopment and basic neuronal functions, consequently showed reduced expression levels. Such abnormalities should be addressed because they might affect unambiguous long-term functionality and hamper the potential of iPSC-derived DA neurons for in vitro disease modeling or cell-based therapy.

## INTRODUCTION

The field of regenerative medicine experienced a powerful impetus after the groundbreaking discovery of induced pluripotency (Takahashi and Yamanaka, 2006). Numerous publications have shown that mouse as well as human induced pluripotent stem cells (iPSCs) have the potency to differentiate into various clinically relevant cell types, such as cardiomyocytes (Kuzmenkin et al., 2009; Ren et al., 2011), hepatocytes (Espejel et al., 2010), hematopoietic progenitors (Hanna et al., 2007), oligodendrocytes (Czepiel et al., 2011), and specific subtypes of neurons (Karumbayaram et al., 2009; Wernig et al., 2008). Such in vitro-generated iPSC-derived cell types provide new possibilities for disease modeling and cell replacement strategies. In particular, the generation of autologous iPSC-derived midbrain dopaminergic (DA) neurons provides a very interesting tool to study and treat Parkinson's disease (PD) (Roessler et al., 2013). However, future clinical application of iPSC-derived DA neurons can only be considered realistic if the desired cell population is strictly purified and completely defined.

Several groups have reported the generation of DA neurons from mouse and human iPSCs (Hargus et al., 2010; Swistowski et al., 2010; Wernig et al., 2008). In these studies, iPSC-derived neurons displayed expression of crucial DA markers and exhibited typical neuronal electrophysiological properties. Furthermore, these iPSC-derived DA neurons could functionally integrate into a rat PD model upon transplantation. In principle, these results indicated that a DA neuronal population could be obtained from iPSCs and present important midbrain DA neuronal characteristics. However, genome-wide studies comparing the genetic and epigenetic features of iPSC-derived DA neurons versus primary DA neurons are currently lacking. Since reprogramming of somatic cells to iPSCs resets their identity back to an embryonic stage, iPSC-derived differentiated neurons should be considered freshly formed "embryonic" neurons. Accordingly, a relevant comprehensive comparison of iPSC-derived DA neurons can only be done with freshly formed embryonic and perinatal primary mesodiencephalic DA (mdDA) neurons.

We generated iPSC lines from *Pitx3*$^{Gfp/+}$ knockin mouse embryonic fibroblasts. PITX3 is a highly specific mdDA neuron marker that is required for DA neuron differentiation in the substantia nigra (Jacobs et al., 2009, 2011; Smidt et al., 2004). Specific PITX3-associated GFP expression

allowed us to strictly identify and purify DA neurons from either iPSCs or the ventral midbrain at specific developmental stages by fluorescence-activated cell sorting (FACS). We then subjected these mdDA neurons to genome-wide gene-expression analysis comparing iPSC-derived DA neurons and primary isolated mdDA neurons.

Induction of pluripotency in somatic cells is considered an epigenetic process that entails, among other events, a large series of changes in DNA methylation patterns (Bock et al., 2011; Maherali et al., 2007; Nishino et al., 2011; Ohi et al., 2011). Furthermore, iPSC differentiation into neural progenitor cells and subsequently into a specific neuronal subtype depends on properly established de novo DNA methylation (Lee et al., 2010; Watanabe et al., 2006). Therefore, it is essential not only to evaluate gene expression but also to compare the methylome of iPSC-derived DA neurons with their primary counterparts. In order to obtain a comprehensive profile of the functionally most relevant DNA methylation sites in iPSC-derived DA neurons versus primary mdDA neurons, we performed a genome-wide analysis of CpG island (CGI) methylation using reduced representation bisulfite sequencing (RRBS) (Meissner et al., 2005; Smallwood et al., 2011) on genomic DNA samples isolated from purified neuronal populations.

## RESULTS

### iPSC-Derived Purified *Pitx3*$^{Gfp/+}$ Neurons Express Crucial DA Markers

iPSCs were generated from embryonic fibroblasts of *Pitx3*$^{Gfp/+}$ transgenic mice and characterized (Figure S1 available online). DA-specific differentiation was performed according to previously described protocols (Chambers et al., 2009; Kawasaki et al., 2000) and resulted in microtubule-associated protein 2 (MAP2)- and tyrosine hydroxylase (TH)-positive PITX3-expressing neurons that exhibited the typical morphology of mature DA neurons (Figures 1A and 1B). A comparative FACS profile showed an absence of GFP-expressing cells within the undifferentiated iPSC population, whereas the DA-differentiated population contained a distinguishable subgroup of GFP-expressing cells (Figure 1C). Upon lineage-specific differentiation, iPSC-derived DA neurons were able to secrete dopamine (Figure 1D), and patch-clamp recordings revealed bona fide electrophysiological properties of the iPSC-derived mdDA neurons (Figures 1E–1G). PITX3-GFP-sorted cells functionally integrated into 6OHDA-lesioned rat brains after intrastriatal implantation, causing a reduction in amphetamine-induced rotation behavior (Figure S2). Our data show successful differentiation of iPSCs toward functional PITX3-expressing mdDA neurons that could be purified from an undefined iPSC-derived cell population. Next, we set out to compare the global gene-expression profile of the iPSC-derived DA neurons with that of the primary mdDA neurons.

### Comparative Expression Profiling: Primary mdDA Neurons versus iPSC-Derived DA Neurons

We performed a genome-wide comparative gene-expression analysis with iPSC-derived PITX3-GFP-positive cells and primary isolated mdDA neurons at several developmental stages (embryonic day 12.5 [E12.5] to postnatal day 0 [P0]; see Figure 2A for experimental scheme). FACS sorting with an efficiency of 98% allowed purification of iPSC-derived PITX3-GFP-positive cells and primary PITX3-GFP-positive mdDA neurons (Figure 2B). Telencephalic brain homogenate served as the negative control for primary cells (Figure 2B), and undifferentiated iPSCs served as the negative control for iPSC-derived GFP-positive cells (as shown in Figure 1C).

Correlation of the genome-wide expression profiles of *Pitx3*$^{Gfp/+}$ mdDA neurons at different developmental stages (Figure 2C) revealed the highest similarities between embryonic mdDA neurons (r = 0.92–0.98). In comparison, the gene-expression profile of iPSC-derived DA neurons was less correlated (highest correlation [r = 0.66] with E14.5 mdDA neurons); however, the weakest correlation was found between iPSC-derived DA neurons and P0 mdDA neurons (r = 0.55).

We performed Gene Ontology (GO) term analyses to annotate genes that were differentially expressed in iPSC-derived DA neurons in comparison with primary embryonic mdDA neurons. We found that the genes that were most downregulated in iPSC-derived DA neurons were associated with GO terms such as nervous system development, neuron differentiation, and neurogenesis (Figure 2D).

We analyzed the expression of an mdDA-specific subset of genes in more detail and found high similarities between iPSC-derived DA neurons and most primary mdDA neurons (Figure 2E). Hierarchical clustering revealed several mdDA-specific genes equally up- or downregulated in primary isolated and iPSC-derived DA neurons. Sample cluster analysis revealed the strongest gene-expression correlation between iPSC-derived mdDA neurons and embryonic primary neurons, whereas postnatal-stage primary mdDA neurons clustered separately. Several key DA genes, such as *Otx2*, *FoxA1*, *FoxA2*, *Nr4a2* (*Nurr1*), *Lmx1a*, and *Lmx1b* were similarly expressed in iPSC-derived mdDA neurons and in embryonic mdDA neurons. Other DA genes, such as *En1* and *En2*, showed expression levels in iPSC-derived mdDA neurons comparable to those in postnatal mdDA neurons. We extended our gene-expression profiling to additional mdDA-specific transcription factors (Chakrabarty et al., 2012), axonal guidance factors, and ion channels (Figure S3). While we observed moderate correlations

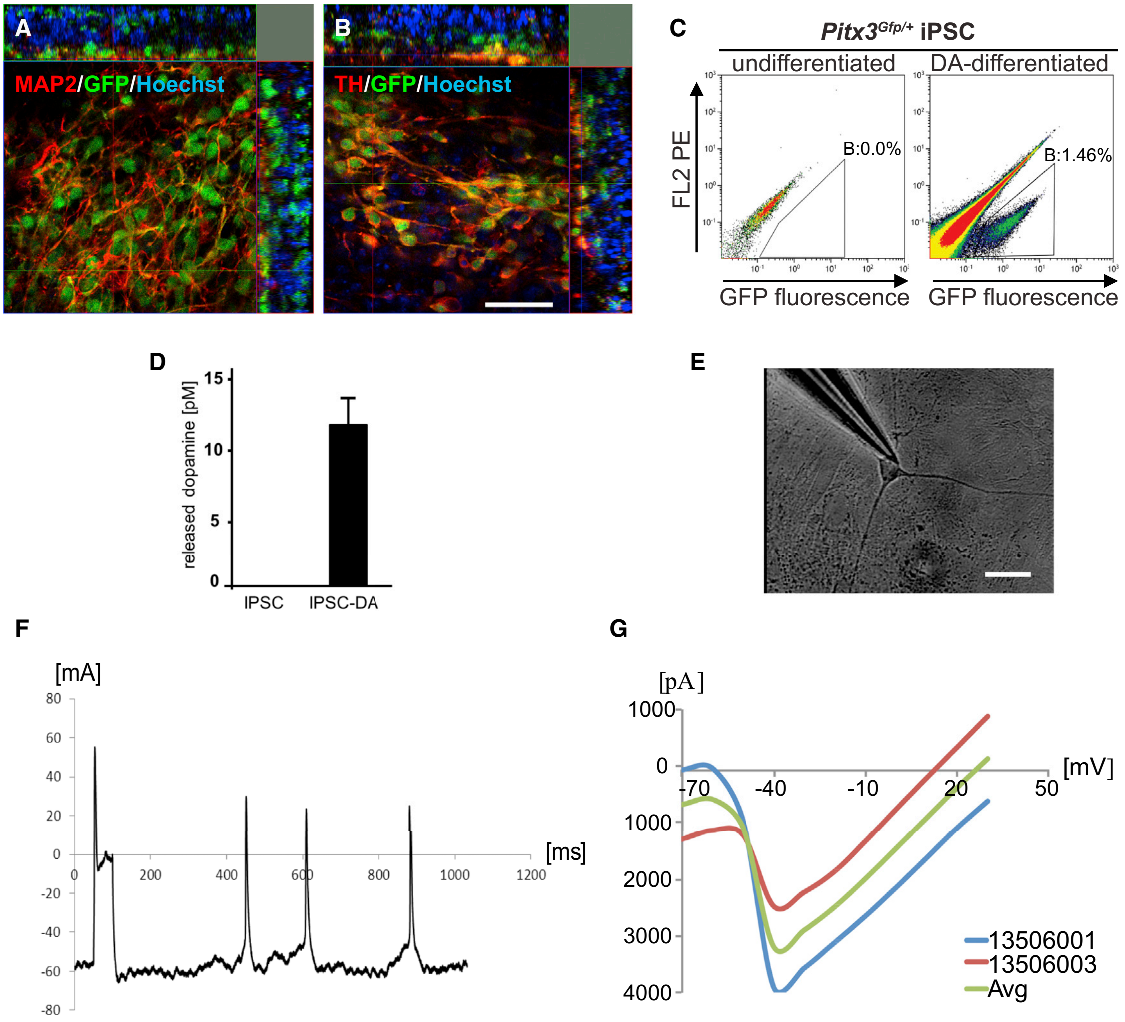


**Figure 1. Characterization of *Pitx3*$^{Gfp/+}$ iPSC-Derived mdDA Neurons**

(A) Confocal image of colabeled MAP2- and GFP-positive cells.

(B) Confocal image of colabeled TH- and GFP-positive cells. Scale bar, 40 μm.

(C) FACS profile comparing undifferentiated and differentiated *Pitx3*$^{Gfp/+}$ iPSCs.

(D) Quantitative dopamine measurement comparing undifferentiated and differentiated *Pitx3*$^{Gfp/+}$ iPSCs (n = 3 independent experiments, error bar represents SD).

(E) Representative phase-contrast image of a recorded neuron. Scale bar, 20 μm.

(F) Current-clamp recording of an in-vitro-generated mdDA neuron.

(G) I-V curve indicative of voltage-independent $Ca^{2+}$ channels and voltage-dependent $Na^{+}$ channels.

See also Figures S1 and S2.

between iPSC-derived mdDA neurons and primary DA neurons for transcription factors and axonal guidance factors, we found a high correlation for expression levels of ion channels, with the strongest overall correlation (r = 0.82) between E12.5 mdDA neurons and iPSC-derived DA neurons.

In view of the origin of iPSC-derived DA neurons, we also analyzed the transcript profile for pluripotency genes and fibroblast-related genes, visualized by dendrograms (Figure 2F). The gene expression of a set of pluripotency markers was subjected to cluster analysis, which showed similar transcript levels (e.g., *Nanog*, *Oct4* (*Pou5f1*), *Zfp42*, and *Nr0b1* [also known as *Dax1*]) in primary and iPSC-derived cell types (Figure 2F). This result not only substantiates a successful transition away from a pluripotent state but also indicates appropriate silencing of pluripotency genes upon in vitro differentiation. However, a similar cluster analysis performed on a subset of

fibroblast-specific markers revealed differential expression in primary and iPSC-derived neurons, suggesting remnants of a still active fibroblast gene program in iPSC-derived DA neurons.

In summary, comparative gene-expression profiling of purified iPSC-derived DA neurons and embryonic mdDA neurons revealed a clear correlation, but less similarity was found between iPSC-derived DA neurons and P0 DA neurons. Downregulated genes in iPSC-derived DA neurons were mainly associated with biological functions such as nervous system development, neurogenesis, and neuron differentiation. These findings prompted us to investigate the nature of this downregulation and to extend our gene-expression analysis by performing in-depth epigenetic profiling focused on DNA methylation.

## Gene-Specific Methylation Profiles Correlate with Gene Expression for Specific DA Markers

First, in order to validate our genome-wide expression profiling data, we performed quantitative PCR (qPCR) experiments for a set of selected key DA genes (*Pitx3*, *Nurr1*, *Dlk1*, *En1*, and *En2*), *Sox9*, and *Desmin*. The expression levels of these particular genes appeared to be in line with the results obtained by microarray analysis, and indicate that DA-specific marker expression in iPSC-derived DA neurons most closely resembles that of terminally differentiated-stage mdDA neurons (Figure 3A). As far as the expression of *Pitx3*, *Nurr1*, *En1*, and *En2* was concerned, the highest similarity was found between iPSC-derived DA and E16.5 mdDA neurons (Figure 3B).

Next, we set out to compare the DNA methylation profiles associated with the selected key DA gene expression of iPSC-derived DA and primary mdDA neurons. DNA methylation is a major player in epigenetic regulation of gene expression, and strong methylation of promoters is generally associated with gene silencing. RRBS allowed us to analyze specific CpG methylation at nucleotide resolution on a genome-wide scale. RRBS provides coverage preferentially of CpG-rich regions, such as CGIs. CGIs are predominantly associated with promoter regions surrounding the transcription start site (TSS). We identified and analyzed CGIs and their methylation status for the aforementioned key mdDA factors and found a striking correlation between CGI methylation levels and gene expression (Figure 3C). *Pitx3*, the mdDA marker we used to purify both cell types, revealed equally low methylation in the CGI around exon 1. Active expression of *Pitx3* in both cell types might be due to this permissive DNA methylation state. CGIs in gene bodies of *Nurr1*, *En1*, and *En2* appeared to be more strongly methylated in iPSC-derived DA neurons than in primary mdDA neurons. This specific DNA methylation state in iPSC-derived DA neurons may underlie the somewhat lower relative levels of expression of these key mdDA factors in iPSC-DA neurons in comparison with the primary mdDA neurons. Interestingly, *Nurr1* showed the highest methylation in a CGI around exon 5, which may point to an alternative promoter for the expression of *Nurr1*. Similarly, increased methylation was observed for *En1* and *En2*, in particular at CGIs around exon 1. Additional key mdDA transcription factors, such as *Otx2*, *Lmx1a*, *Lmx1b*, *FoxA1*, *FoxA2*, and the dopamine receptor *Drd2*, were analyzed and their CGI methylation status was examined (Figure S4). Overall, increased CGI methylation in iPSC-DA neurons appeared to be at intermediate levels (around 50%) in comparison with native mdDA neurons. These differences in DNA methylation patterns and, accordingly, the reduction in DA gene-expression levels may have subtle, as yet uncharacterized effects on the proper functionality of iPSC-derived DA neurons. In order to obtain a global view of DNA methylation, we next set out to compare total CpG methylation and CGI methylation in primary mdDA neurons and iPSC-derived mdDA neurons.

## DNA Methylation Landscape in iPSC-Derived DA Neurons and Primary mdDA Neurons

To determine whether DA neurons derived from iPSCs recapitulate the DNA methylation landscape of their in vivo counterparts isolated from the ventral midbrain, we performed a genome-wide analysis of 5mC (Figure 4). We obtained information on 844,812 CpGs ($\geq$5 read depth) for iPSC-DA neurons and mdDA neurons, with a bisulfite conversion efficiency of >98% as assessed by non-CpG methylation. Of these, 72% corresponded to CGIs in iPSC-DA neurons and mdDA neurons. Global methylation levels outside of CGIs were similar in iPSC-DA and mdDA neurons, and comparable to those observed in somatic tissues in general (Illingworth et al., 2008, 2010; Figures 4A and 4B). Similarly, the CpG methylation levels of iPSC-DA neurons and mdDA neurons were highly correlated (r = 0.85; Figures 4C and 4D). On the other hand, in a CGI context, iPSC-DA neurons were relatively hypermethylated in comparison with mdDA neurons (Figure 4B), with the relative proportion of CpGs presenting intermediate levels of methylation (i.e., 40%–60%; Figures 4B and 4C). By plotting the percentage of CGI methylation in mdDA neurons against iPSC-DA neurons, we found a population of more than 2,000 genes with relative CGI-associated hypermethylation in iPSC-DA neurons (Figure 4D). As described above, this was also observed in the methylation levels of individual CGIs, with CGIs unmethylated in mdDA neurons presenting intermediate levels of methylation in iPSC-DA neurons (Figure 3C). Since CGI methylation is essentially bimodal (i.e., either methylated or unmethylated), this reflects a degree of epigenetic heterogeneity within the population of iPSC-derived DA neurons. To

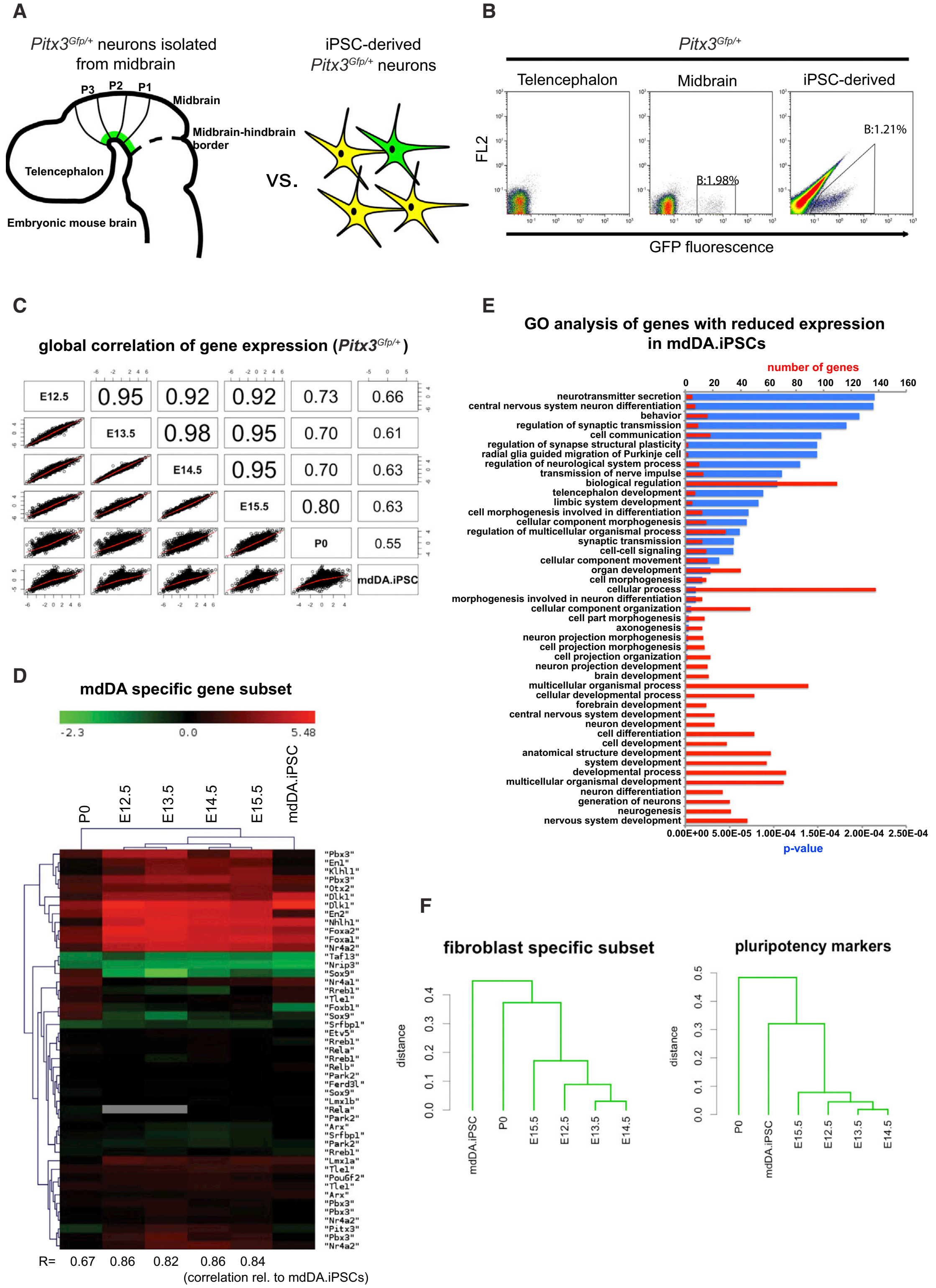


**Figure 2. Gene-Expression Profiling: Mesodiencephalic PITX3+ Neurons versus iPSC-Derived PITX3+ Neurons**

(A) Schematic of the experimental setup.

(B) FACS profile of the telencephalon and midbrain of *Pitx3*$^{Gfp/+}$ mice (E14.5) compared with iPSC-derived PITX3+ neurons.

(C) Correlation matrix of global gene expression comparing all *Pitx3*$^{Gfp/+}$ purified neurons.

*(legend continued on next page)*

assess whether there is an impact on gene expression in this distinctly hypermethylated population, we analyzed the function of the associated genes and compared expression levels in developing mdDA neurons and iPSC-DA neurons.

### Intermediate Hypermethylation in iPSC-DA Neurons Affects Cell-Type-Specific Gene Expression

Although gene expression in general did not seem to be reduced in iPSC-derived DA neurons, a significant proportion of hypermethylated CGIs was observed (Figures 4D and S5). The most hypermethylated subset of genes (Figure S5C) was subjected to GO analysis (Figure 5A). Interestingly, the most significantly enriched biological functions, such as neuron differentiation and neuron development, corresponded with the most enriched GO terms found in our reduced-gene-expression analysis (see Figure 2D). We then analyzed the correlation of gene expression for genes involved in neuron differentiation (169 genes with a corrected p value of 2.93E-47) between all developmental stages and iPSC-derived DA neurons (Figure 5B). Correlations of neuron differentiation-associated gene expression in iPSC-derived neurons strongly decreased with increasing maturation of the embryonic mdDA neurons, with a correlation coefficient as low as 0.28 for iPSC-derived DA neurons versus P0 mdDA neurons. Of note, we found a higher correlation of gene expression between prenatal mdDA neurons and iPSC-derived neurons than between prenatal mdDA neurons and P0 mdDA neurons.

After defining the biological functions of the hypermethylated subgroup of genes and establishing the correlation of expression for genes in the GO group "neuron differentiation," we revisited the gene-expression data to visualize the actual transcript levels within a selection of the GO terms (Figures 5C–5E). We found substantial downregulation for multiple genes in three representative GO groups: "neuron development," "axon guidance," and "inner ear development" (nonneural GO term). Large clusters of downregulated genes were predominantly observed in P0 mdDA neurons and iPSC-derived DA neurons.

Thus, we identified a subset of genes in iPSC-derived DA neurons that show intermediate hypermethylation. Many, but not all, of these genes show reduced expression levels compared with embryonic mdDA neurons. Crucially, gene annotation revealed that a large fraction of the hypermethylated genes are involved in biological functions relevant to neuron development and differentiation.

## DISCUSSION

In view of their potential use in cell-based therapy for patients with PD, midbrain DA neurons were one of the first cell types to be generated from iPSCs. The resemblance between iPSC-derived DA neurons and true DA neurons has been studied based on a number of mainly morphological and functional properties, as well as mdDA-specific gene sets (Hedlund et al., 2008; Kriks et al., 2011; Wernig et al., 2008). However, the risk of tumor growth and the heterogeneous molecular backgrounds of embryonic stem cell (ESC)- and iPSC-derived DA neurons have precluded the use of these cells for therapy in humans (Momčilović et al., 2014; Salti et al., 2013). Gaining a detailed understanding of the genetic and epigenetic signatures of ESC- and iPSC-derived DA neurons is therefore a critical step toward establishing cell-based therapy as a viable treatment for PD. In this study, we present a comparative genome-wide profile of the genetic and epigenetic features of iPSC-derived DA neurons and their primary counterparts. Although iPSC-derived DA neurons showed characteristics that are widely used to classify functional mdDA neurons (Ganat et al., 2012; Kriks et al., 2011; Sundberg et al., 2013), caused a reduction in rotation behavior in a rat model for PD, and adopted many of the genetic and epigenetic features of their primary counterparts, we also observed major deviations that may interfere with proper functionality after grafting.

Since DA neurons obtained via induced pluripotency undergo defined developmental stages in vitro, we compared them with primary embryonic and postnatal mdDA neurons. To generate iPSCs and differentiate them into DA neurons, we applied a combination of currently widely accepted and standardized protocols (Chambers et al., 2009; Kawasaki et al., 2000; Takahashi and Yamanaka, 2006). We made use of transgenic $Pitx3^{Gfp/+}$ mice (Maxwell et al., 2005) to isolate primary mdDA neurons and reprogram embryonic fibroblasts into iPSCs, which were subsequently differentiated into DA neurons. The transcription factor PITX3 has been demonstrated to be one of the most stringent markers for fully differentiated, functional mdDA neurons (Smidt et al., 2003, 2004), and expression of the PITX3-GFP reporter allowed us to strictly identify and FACS purify iPSC-derived and primary mdDA neurons.

Our global gene-expression analysis showed that iPSC-derived mdDA neurons were highly similar to embryonic primary mdDA neurons, particularly when we focused on

(D) GO term analysis of genes that were most reduced in iPSC-derived mdDA neurons.
(E) Expression levels of selected mdDA-specific genes, comparing developmental midbrain stages with iPSC-derived PITX3+ neurons. R, correlation coefficient relative to iPSC-derived DA neurons.
(F) Hierarchical clustering for a subset of pluripotency and fibroblast markers.
See also Figure S3.

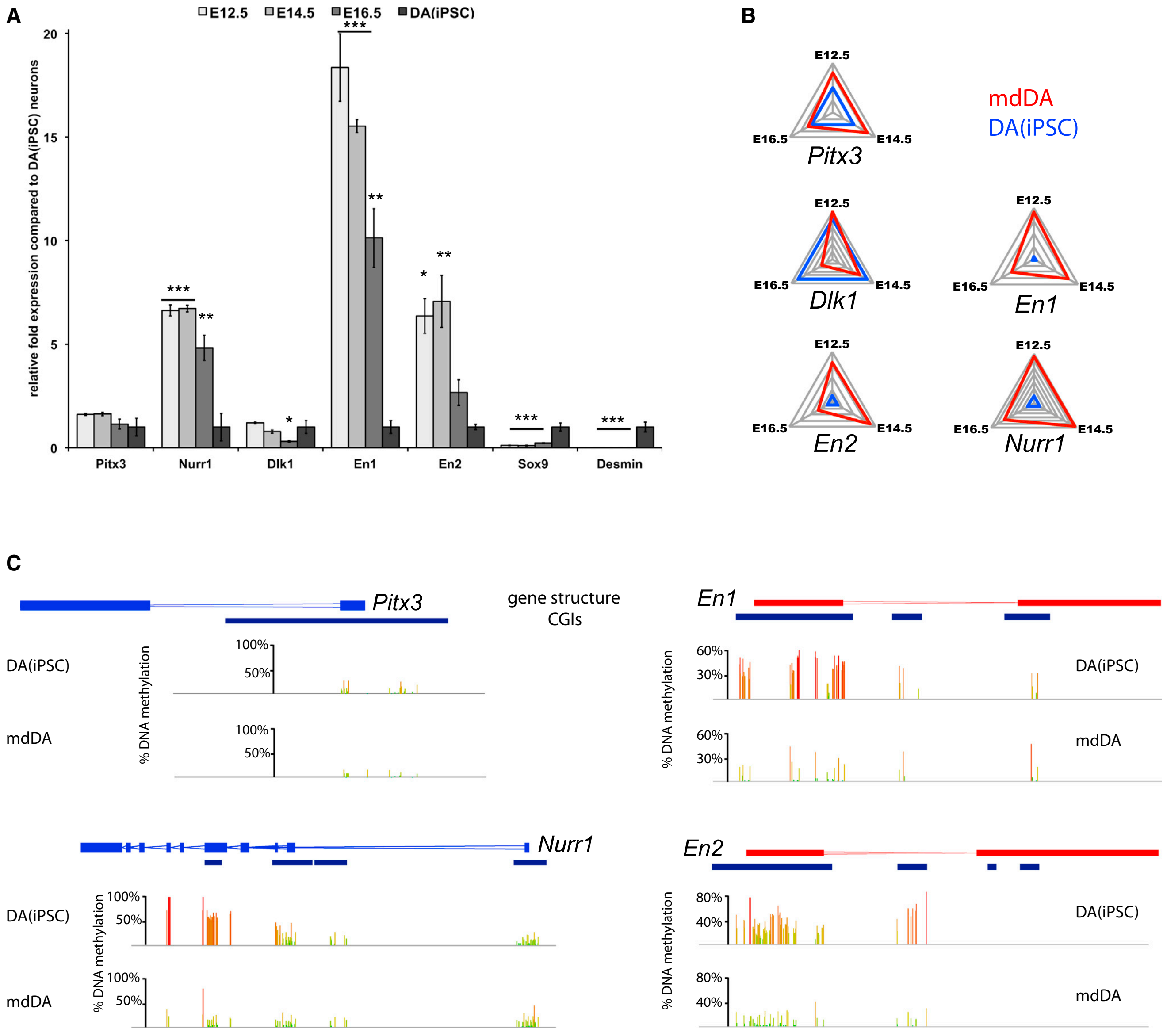


**Figure 3. Gene-Specific Methylation Profiles Correlate with Gene Expression for Specific DA Markers**

(A) qPCR profile comparing the transcript levels of *Pitx3*, *Nurr1*, *Dlk1*, *En1/2*, *Sox9*, and *desmin* in embryonic mdDA neurons and iPSC-derived DA neurons (both purified on $Pitx3^{Gfp/+}$ expression). One-way ANOVA: ***$p < 0.001$; **$p < 0.01$; *$p < 0.05$; $n = 3$ (biological replicates); error bars represent SDs around the mean.

(B) Net plots for *Pitx3*, *Nurr1*, *En1*, *En2*, and *Dlk1* indicate the closest similarities between E16.5 mdDA neurons and iPSC-derived DA neurons for most markers. Axes represent relative fold expression and similarity in primary mdDA neurons (E12.5, E14.5, and E15.5) versus iPSC-DA neurons.

(C) Gene-specific DNA methylation profiling reveals nearly identical methylation status for the *Pitx3* gene in primary mdDA neurons and iPSC-derived DA neurons. For *Nurr1* and *En1/2*, intermediate (~50%) hypermethylation was found. Hypermethylated gene iPSC-DA neurons show decreased transcript levels compared with mdDA neurons. Methylation intensities across CGIs are represented in % (red bars, high CpG methylation; yellow bars, intermediate CpG methylation; green bars, low CpG methylation).

See also Figure S4.

mdDA-specific genes. However, we identified a subset of genes that were downregulated in iPSC-derived mdDA neurons in comparison with embryonic primary mdDA neurons. GO analysis revealed that these genes were linked to terms such as nervous system development, neuron differentiation, and neurogenesis.

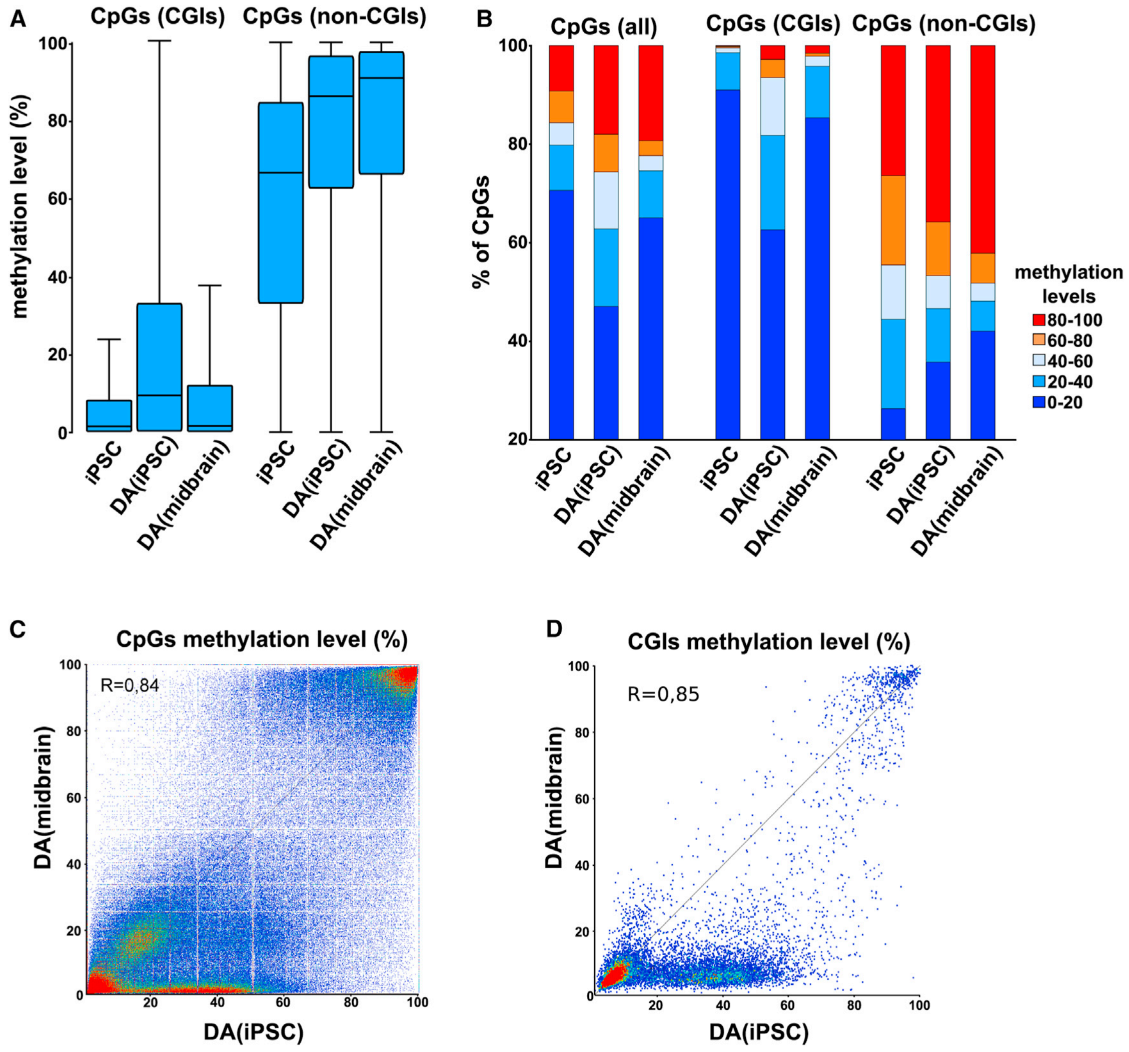


**Figure 4. Global Comparative Methylation Profile of iPSC-DA Neurons versus mdDA Neurons**

(A) Boxplots representing the range of methylation of CGIs and non-CGIs in iPSCs, iPSC-DA neurons, and mdDA neurons. Error bars represent minimum and maximum values.

(B) Distribution of CpG methylation levels across the genome in CGIs and non-CGI context in iPSCs, iPSC-DA neurons, and mdDA neurons.

(C) Correlation of genome-wide CpG methylation in iPSC-DA neurons versus mdDA neurons.

(D) Correlation of global CGI methylation in iPSC-DA neurons versus mdDA neurons.

See also Figure S5.

Although transcript analysis indicated that proviral gene expression introduced during reprogramming was silenced in iPSC-derived mdDA neurons, we still found residual expression of fibroblast markers. It was previously shown that iPSCs are prone to differentiate along their somatic parental lineages because they maintain a parental epigenetic memory (Bar-Nur et al., 2011; Kim et al., 2010; Sullivan et al., 2010). Although some studies suggested that such epigenetic memory is restricted to early-passage iPSCs (Polo et al., 2010), others reported that parental epigenetic states also persist in late-passage iPSCs (Kim et al., 2011). This could explain why we observed fibroblast marker expression and a permissive DNA methylation state for some of the fibroblast genes in PITX3-GFP-positive cells derived from high-passage (p15–p20) iPSCs.

To determine whether the epigenetic states of the analyzed neuron populations reflected similarities and differences in gene-expression profiles, we analyzed global DNA methylation patterns in primary embryonic mdDA neurons (E14.5) and iPSC-derived DA neurons. In contrast to the case with undifferentiated iPSCs, we found strong general de novo methylation in iPSC-derived DA neurons.

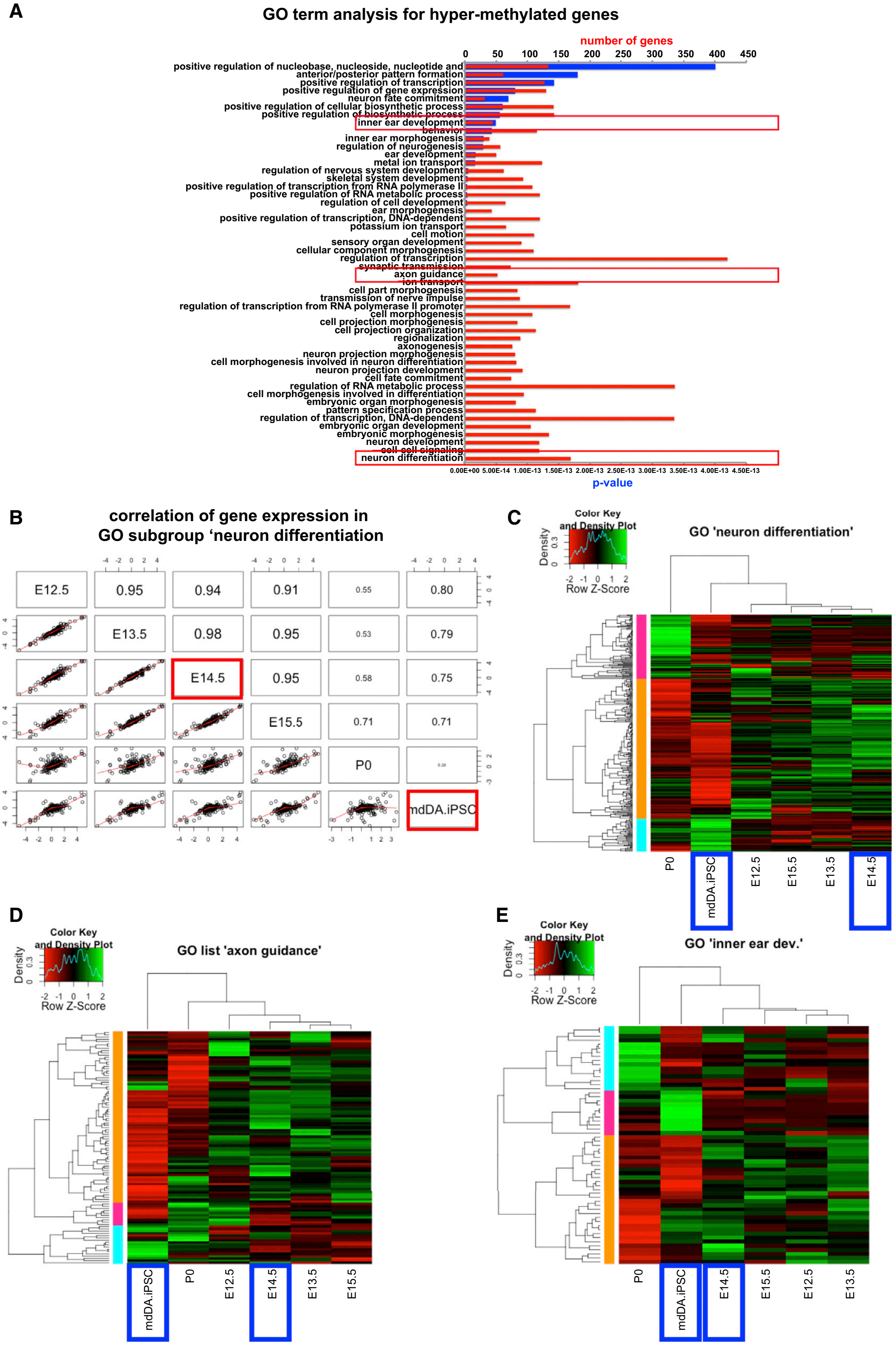


*(legend on next page)*

Earlier studies reported that PSCs mostly contain methylation-free promoters, as well as methylation-free intergenic and orphan CGIs (Illingworth et al., 2010). De novo methylation was only found upon loss of pluripotency, suggesting that transcriptional repression in PSCs is established predominantly via other mechanisms (Fouse et al., 2008; Mohn et al., 2008). Indeed, we observed that most de novo methylation events occurred during differentiation from pluripotent cells to multipotent precursors isolated from an iPSC-derived NESTIN-GFP reporter line (data not shown).

General methylation states appeared to be comparable between iPSC-derived DA neurons and primary embryonic mdDA neurons (r = 0.85), indicating that our in-vitro-generated DA neurons widely adopted the epigenetic signature of their primary counterparts. Nonetheless, we found several thousand genes hypermethylated in iPSC-derived DA neurons. This hypermethylation was found predominantly at an intermediate level ranging from 40% to 60% DNA methylation. At this point, we can only speculate about whether hypermethylation is due to retained epigenetic memory or to heterogeneous differentiation/maturation stages within the PITX3-GFP FACS-sorted iPSC-derived population. To our knowledge, a specific neuronal subtype derived from iPSCs has not been characterized to such an extent, but similar differences in methylation profiles have been observed in reprogrammed mesenchymal stromal cells compared with ESCs (Shao et al., 2013). Interestingly, our gene-expression data show reduced expression of *Tet1* and *Tet3* in iPSC-derived DA neurons compared with E14.5 mdDA neurons. Tet proteins have been shown to be crucial for establishment of pluripotency, development, and neuronal activity (Koh et al., 2011; Rudenko et al., 2013; Zhang et al., 2013). Presently, very little is known about the activity of Tet proteins in specific subtypes of neurons (primary or PSC derived). It is becoming more and more clear, however, that their demethylation activity is crucial for functionally bona fide, healthy neurons (Gavin et al., 2013; Ma et al., 2009). Our DNA methylation profiling of iPSC-derived DA neurons versus primary mdDA neurons did not allow a distinction between 5-methylcytosine (5mC) and 5-hydroxymethylcytosine (5hmC). Neuronal gene activation was recently shown to be mediated by specific MeCP2 binding to 5hmC (Li et al., 2013; Mellén et al., 2012). Therefore, we cannot rule out the possibility that elevated 5hmC levels in iPSC-derived mdDA neurons might contribute to gene activation rather than to silencing. However, within the population of hypermethylated genes, we did observe gene clusters with substantially lower transcript levels. Interestingly, we also found clusters of genes that were upregulated compared with primary mdDA neurons. Because these genes also have been identified by RRBS based on their intermediate hypermethylation, these particular subsets might reflect groups of genes that are activated rather than silenced upon 5-hydroxymethylation.

In our gene-expression analyses, we found a weaker correlation between iPSC-derived mdDA neurons and P0 primary mdDA neurons than between iPSC-derived mdDA neurons and embryonic primary mdDA neurons, which prompted us to analyze in depth the underlying methylation states of only E14.5 mdDA neurons and iPSC-derived mdDA neurons. Interestingly, however, the expression levels of *En1* and *En2* in iPSC-derived mdDA neurons were comparable to those in P0 primary mdDA neurons. The meaning of this in terms of functionality is unclear and may reflect the fact that these iPSC-derived mdDA neurons are in more of an adult state with respect to *En1* and *En2*. In addition, it might indicate that iPSC-derived mdDA neurons are in an A9 state, as it has been suggested that *En1* levels are downregulated by PITX3 in the rostral mdDA subpopulation (Veenvliet et al., 2013).

Our findings raise two important questions: is epigenetic memory an obstacle for exploiting the full potential of iPSCs (e.g., personalized disease modeling), and do aberrations in the expression profiles of iPSC-derived mdDA neurons interfere with long-term functionality? Although comprehensive studies have been performed to test the in vivo functionality of human neurons (Ganat et al., 2012; Kriks et al., 2011), these questions still need to be addressed, especially when in-vitro-generated cells are to be considered for application in disease modeling and cell-based therapy for PD. It remains to be studied whether modifications in the reprogramming process, as recently reported for the human system (Gafni et al., 2013), as well as refined differentiation procedures might diminish these deviations. An increased fundamental understanding of the genetic and epigenetic signatures of DA neurons in vivo (Hegarty et al., 2013; Smidt and Burbach, 2007; van Heesbeen et al., 2013) could offer new leads to generate safe and transplantable DA neurons in vitro.

**Figure 5. Intermediate Hypermethylation of >2,000 Genes in iPSC-Derived DA Neurons Affects Cell-Type-Specific Gene Expression**
(A) GO term analysis of hypermethylated genes in iPSC-derived DA neurons.
(B) Correlation matrix for gene expression in the "neuron differentiation" GO subset (highlighted by the lowest red box in A).
(C–E) Heatmaps showing comparative gene expression for genes involved in neuron differentiation (C), axon guidance (D), and inner ear development (E) (GO terms highlighted by red boxes in A). Blue boxes indicate the groups used for RRBS analysis.

## EXPERIMENTAL PROCEDURES

### Mice

*Pitx3(Gfp/+)* embryos at several developmental stages were obtained by intercrossing C57BL6/J with *Pitx3(Gfp/Gfp)* mice. *Pitx3(Gfp/+)* embryos are heterozygous for wild-type PITX3 and have normal mdDA system development (Maxwell et al., 2005). Overlap of endogenous PITX3 with GFP has been shown to be ~100% (Maxwell et al., 2005). All procedures were approved by and performed according to the guidelines of the Dutch ethics committees for animal experiments (UMCU and UvA).

### iPSC Generation and Propagation

Mouse embryonic fibroblasts were isolated from E14.5 embryos of $Pitx3^{Gfp/+}$ and *Nestin-Gfp* mice, both of which were previously described and characterized (Yamaguchi et al., 2000; Zhao et al., 2004). Fibroblasts were cultured until passage 5–8 and then retrovirally transfected with the four Yamanaka reprogramming factors. Separate vectors containing either *Oct4*, *Klf4*, *Sox2*, or *cMyc* were used for pluripotency induction. Retroviruses were obtained from Phoenix Eco packaging cells transfected with the reprogramming factors (for vector information: Addgene, http://www.addgene.org). The detailed induction protocol was previously described (Czepiel et al., 2011). iPSC clones were characterized by immunocytochemistry, RT-PCR, western blot, and bisulfite sequencing for the promoter regions of *Nanog* and *Oct4* (Figure S1).

### Differentiation of $Pitx3^{Gfp/+}$ iPSCs toward DA Neurons

We combined a stromal feeder-based protocol (Kawasaki et al., 2000) with dual bone morphogenetic protein (BMP) and transforming growth factor β (TGF-β) inhibition (Chambers et al., 2009). iPSC colonies previously cultured on gelatin were manually picked and seeded on MS5 stromal cells in serum replacement medium containing Dulbecco's modified Eagle's medium F12 (DMEM-F12), 15% knockout serum, glutamate, and β-mercaptoethanol. The cells were allowed to settle for 24 hr, and then Noggin (300 ng/ml) and the TGF-β inhibitor SB431541 (10 μM) were added for 4–5 days. Neural precursors (NESTIN-GFP) were collected after 14 days of differentiation. The medium was gradually changed to N2 containing Sonic hedgehog (SHH; 200 ng/ml). After neural induction, neuronal patterning and DA differentiation were induced using a combination of brain-derived neurotrophic factor (BDNF), ascorbic acid, SHH, and fibroblast growth factor 8 (FGF8) in N2 medium. Maturation was initiated by withdrawing SHH and FGF8 in the presence of BDNF, glial cell-derived neurotrophic factor (GDNF), ascorbic acid, and cyclic adenosine monophosphate (Perrier et al., 2004). At about 4 weeks of differentiation, $Pitx3^{Gfp/+}$ neurons reached a mature state as determined by morphology, marker expression, electrophysiology, and dopamine production. Continuous culturing in these conditions did not induce any further maturation/differentiation, i.e., there were no changes in cell morphology, level and profile of marker expression, electric membrane properties, or dopamine production; however, continuous culturing resulted in an increase in cell death. For gene expression and DNA methylation analyses, we used $Pitx3^{Gfp/+}$ mdDA neurons derived from iPSCs after 4 weeks of differentiation. Cells were sorted and immediately subjected to RNA or DNA isolation.

### Microarray Analysis

Total RNA was isolated from embryonic ventral midbrain tissue at various developmental stages (four biological replicates each) and iPSC-derived neurons (three biological replicates) using Trizol (Invitrogen), and purified using RNeasy columns (QIAGEN). Microarray analysis was performed in biological triplicates. For each experimental sample, a dye swap was performed to correct for dye effects. Agilent whole mouse genome microarray (G4122F; Agilent) sets were used for all hybridization. The array set is comprised of 60-mer oligonucleotide probes representing over 41,000 mouse genes and transcripts. Hybridized slides were scanned on an Agilent scanner (G2565AA) at 100% laser power, 30% PMT. After data were extracted using ImaGene 8.0 (BioDiscovery), print-tip Loess normalization was performed on mean spot intensities. Data were analyzed using ANOVA (R version 2.2.1/MAANOVA version 0.98-7; http://www.r-project.org/), and p values were determined by a permutation F2 test in which residuals were shuffled 5,000 times globally.

### RRBS

RRBS was performed as described previously (Smallwood et al., 2011). Genomic DNA from E14.5 mdDA neurons, NESTIN-GFP-positive precursors, and iPSC-derived DA neurons was purified using the QIAamp Micro Kit (QIAGEN) followed by MspI digestion (Fermentas), end-repair/A-tailing, and ligation (T4 Ligase, Fermentas) of 5mC-adapters (Illumina) performed in Tango1X buffer without intermediate purification of the enzymatic reactions (heat inactivation was used after each enzymatic step and components were adjusted for the next step). Bisulfite conversion was performed (Imprint DNA modification; Sigma) and converted DNA was amplified (six cycles) using uracil stalling free polymerase (Pfu Turbo Cx; Stratagene) followed by size selection (150–450 bp, Qiaquick; QIAGEN) and a second round of amplification (10–12 cycles, Platinium, Pfx polymerase; Invitrogen). Libraries were purified (SPRI beads; Agencourt) and sequenced on an Illumina Genome Analyzer IIx (40 bp, single read). Quality control of raw sequence data was performed using FastQC (http://www.bioinformatics.babraham.ac.uk/projects/fastqc), and trimming was done using TrimGalore (http://www.bioinformatics.babraham.ac.uk/projects/trim_galore/). Sequence alignment and methylation calls were performed using Bareback (http://www.bioinformatics.babraham.ac.uk/projects/bareback/) and Bismark (Krueger and Andrews, 2011)

## ACCESSION NUMBERS

Data for the expression arrays (KC002) reported here have been deposited in the GEO database under accession number GSE47178. The RRBS data have been deposited in the GEO database under accession number GSE55475.

## SUPPLEMENTAL INFORMATION

Supplemental Information includes Supplemental Experimental Procedures, five figures, and one table and can be found

with this article online at http://dx.doi.org/10.1016/j.stemcr.2014.03.001.

## ACKNOWLEDGMENTS

We thank N. Brouwer and M. Meijer for their technical support and F. Krueger (Babraham Institute) for assisting with the bioinformatics. We acknowledge the help of the FACS core facility of UMCG and thank R. Wichmann for performing animal surgeries. R.R. was supported by the Hazewinkel-Beringer Foundation and the Jan Kornelius de Cock Stichting. Confocal imaging was performed at the UMCG Microscopy and Imaging Center (UMIC) and sponsored by NWO grants 40-00506-98-9021 and 175010-2009-23. This work was supported by a VICI grant (No. 865.09.002) to M.P.S. Work in G.K.'s lab is supported by the Biotechnology and Biological Sciences Research Council and Medical Research Council of the UK.

Received: June 21, 2013
Revised: March 4, 2014
Accepted: March 5, 2014
Published: April 3, 2014

## REFERENCES

Bar-Nur, O., Russ, H.A., Efrat, S., and Benvenisty, N. (2011). Epigenetic memory and preferential lineage-specific differentiation in induced pluripotent stem cells derived from human pancreatic islet beta cells. Cell Stem Cell *9*, 17–23.

Bock, C., Kiskinis, E., Verstappen, G., Gu, H., Boulting, G., Smith, Z.D., Ziller, M., Croft, G.F., Amoroso, M.W., Oakley, D.H., et al. (2011). Reference Maps of human ES and iPS cell variation enable high-throughput characterization of pluripotent cell lines. Cell *144*, 439–452.

Chakrabarty, K., Von Oerthel, L., Hellemons, A., Clotman, F., Espana, A., Groot Koerkamp, M., Holstege, F.C., Pasterkamp, R.J., and Smidt, M.P. (2012). Genome wide expression profiling of the mesodiencephalic region identifies novel factors involved in early and late dopaminergic development. Biol. Open *1*, 693–704.

Chambers, S.M., Fasano, C.A., Papapetrou, E.P., Tomishima, M., Sadelain, M., and Studer, L. (2009). Highly efficient neural conversion of human ES and iPS cells by dual inhibition of SMAD signaling. Nat. Biotechnol. *27*, 275–280.

Czepiel, M., Balasubramaniyan, V., Schaafsma, W., Stancic, M., Mikkers, H., Huisman, C., Boddeke, E., and Copray, S. (2011). Differentiation of induced pluripotent stem cells into functional oligodendrocytes. Glia *59*, 882–892.

Espejel, S., Roll, G.R., McLaughlin, K.J., Lee, A.Y., Zhang, J.Y., Laird, D.J., Okita, K., Yamanaka, S., and Willenbring, H. (2010). Induced pluripotent stem cell-derived hepatocytes have the functional and proliferative capabilities needed for liver regeneration in mice. J. Clin. Invest. *120*, 3120–3126.

Fouse, S.D., Shen, Y., Pellegrini, M., Cole, S., Meissner, A., Van Neste, L., Jaenisch, R., and Fan, G. (2008). Promoter CpG methylation contributes to ES cell gene regulation in parallel with Oct4/Nanog, PcG complex, and histone H3 K4/K27 trimethylation. Cell Stem Cell *2*, 160–169.

Gafni, O., Weinberger, L., Mansour, A.A., Manor, Y.S., Chomsky, E., Ben-Yosef, D., Kalma, Y., Viukov, S., Maza, I., Zviran, A., et al. (2013). Derivation of novel human ground state naive pluripotent stem cells. Nature *504*, 282–286.

Ganat, Y.M., Calder, E.L., Kriks, S., Nelander, J., Tu, E.Y., Jia, F., Battista, D., Harrison, N., Parmar, M., Tomishima, M.J., et al. (2012). Identification of embryonic stem cell-derived midbrain dopaminergic neurons for engraftment. J. Clin. Invest. *122*, 2928–2939.

Gavin, D.P., Chase, K.A., and Sharma, R.P. (2013). Active DNA demethylation in post-mitotic neurons: a reason for optimism. Neuropharmacology *75*, 233–245.

Hanna, J., Wernig, M., Markoulaki, S., Sun, C.W., Meissner, A., Cassady, J.P., Beard, C., Brambrink, T., Wu, L.C., Townes, T.M., and Jaenisch, R. (2007). Treatment of sickle cell anemia mouse model with iPS cells generated from autologous skin. Science *318*, 1920–1923.

Hargus, G., Cooper, O., Deleidi, M., Levy, A., Lee, K., Marlow, E., Yow, A., Soldner, F., Hockemeyer, D., Hallett, P.J., et al. (2010). Differentiated Parkinson patient-derived induced pluripotent stem cells grow in the adult rodent brain and reduce motor asymmetry in Parkinsonian rats. Proc. Natl. Acad. Sci. USA *107*, 15921–15926.

Hedlund, E., Pruszak, J., Lardaro, T., Ludwig, W., Viñuela, A., Kim, K.S., and Isacson, O. (2008). Embryonic stem cell-derived Pitx3-enhanced green fluorescent protein midbrain dopamine neurons survive enrichment by fluorescence-activated cell sorting and function in an animal model of Parkinson's disease. Stem Cells *26*, 1526–1536.

Hegarty, S.V., Sullivan, A.M., and O'Keeffe, G.W. (2013). Midbrain dopaminergic neurons: a review of the molecular circuitry that regulates their development. Dev. Biol. *379*, 123–138.

Illingworth, R., Kerr, A., Desousa, D., Jørgensen, H., Ellis, P., Stalker, J., Jackson, D., Clee, C., Plumb, R., Rogers, J., et al. (2008). A novel CpG island set identifies tissue-specific methylation at developmental gene loci. PLoS Biol. *6*, e22.

Illingworth, R.S., Gruenewald-Schneider, U., Webb, S., Kerr, A.R., James, K.D., Turner, D.J., Smith, C., Harrison, D.J., Andrews, R., and Bird, A.P. (2010). Orphan CpG islands identify numerous conserved promoters in the mammalian genome. PLoS Genet. *6*, e1001134.

Jacobs, F.M., van Erp, S., van der Linden, A.J., von Oerthel, L., Burbach, J.P., and Smidt, M.P. (2009). Pitx3 potentiates Nurr1 in dopamine neuron terminal differentiation through release of SMRT-mediated repression. Development *136*, 531–540.

Jacobs, F.M., Veenvliet, J.V., Almirza, W.H., Hoekstra, E.J., von Oerthel, L., van der Linden, A.J., Neijts, R., Koerkamp, M.G., van Leenen, D., Holstege, F.C., et al. (2011). Retinoic acid-dependent and -independent gene-regulatory pathways of Pitx3 in meso-diencephalic dopaminergic neurons. Development *138*, 5213–5222.

Karumbayaram, S., Novitch, B.G., Patterson, M., Umbach, J.A., Richter, L., Lindgren, A., Conway, A.E., Clark, A.T., Goldman, S.A., Plath, K., et al. (2009). Directed differentiation of human-induced pluripotent stem cells generates active motor neurons. Stem Cells *27*, 806–811.

Kawasaki, H., Mizuseki, K., Nishikawa, S., Kaneko, S., Kuwana, Y., Nakanishi, S., Nishikawa, S.I., and Sasai, Y. (2000). Induction of

midbrain dopaminergic neurons from ES cells by stromal cell-derived inducing activity. Neuron *28*, 31–40.

Kim, K., Doi, A., Wen, B., Ng, K., Zhao, R., Cahan, P., Kim, J., Aryee, M.J., Ji, H., Ehrlich, L.I.R., et al. (2010). Epigenetic memory in induced pluripotent stem cells. Nature *467*, 285–290.

Kim, K., Zhao, R., Doi, A., Ng, K., Unternaehrer, J., Cahan, P., Huo, H., Loh, Y.-H., Aryee, M.J., Lensch, M.W., et al. (2011). Donor cell type can influence the epigenome and differentiation potential of human induced pluripotent stem cells. Nat. Biotechnol. *29*, 1117–1119.

Koh, K.P., Yabuuchi, A., Rao, S., Huang, Y., Cunniff, K., Nardone, J., Laiho, A., Tahiliani, M., Sommer, C.A., Mostoslavsky, G., et al. (2011). Tet1 and Tet2 regulate 5-hydroxymethylcytosine production and cell lineage specification in mouse embryonic stem cells. Cell Stem Cell *8*, 200–213.

Kriks, S., Shim, J.-W., Piao, J., Ganat, Y.M., Wakeman, D.R., Xie, Z., Carrillo-Reid, L., Auyeung, G., Antonacci, C., Buch, A., et al. (2011). Dopamine neurons derived from human ES cells efficiently engraft in animal models of Parkinson's disease. Nature *480*, 547–551.

Krueger, F., and Andrews, S.R. (2011). Bismark: a flexible aligner and methylation caller for Bisulfite-Seq applications. Bioinformatics *27*, 1571–1572.

Kuzmenkin, A., Liang, H., Xu, G., Pfannkuche, K., Eichhorn, H., Fatima, A., Luo, H., Saric, T., Wernig, M., Jaenisch, R., and Hescheler, J. (2009). Functional characterization of cardiomyocytes derived from murine induced pluripotent stem cells in vitro. FASEB J. *23*, 4168–4180.

Lee, S.-H., Jeyapalan, J.N., Appleby, V., Mohamed Noor, D.A., Sottile, V., and Scotting, P.J. (2010). Dynamic methylation and expression of Oct4 in early neural stem cells. J. Anat. *217*, 203–213.

Li, Y., Wang, H., Muffat, J., Cheng, A.W., Orlando, D.A., Lovén, J., Kwok, S.M., Feldman, D.A., Bateup, H.S., Gao, Q., et al. (2013). Global transcriptional and translational repression in human-embryonic-stem-cell-derived Rett syndrome neurons. Cell Stem Cell *13*, 446–458.

Ma, D.K., Jang, M.-H., Guo, J.U., Kitabatake, Y., Chang, M.-L., Pow-Anpongkul, N., Flavell, R.A., Lu, B., Ming, G.-L., and Song, H. (2009). Neuronal activity-induced Gadd45b promotes epigenetic DNA demethylation and adult neurogenesis. Science *323*, 1074–1077.

Maherali, N., Sridharan, R., Xie, W., Utikal, J., Eminli, S., Arnold, K., Stadtfeld, M., Yachechko, R., Tchieu, J., Jaenisch, R., et al. (2007). Directly reprogrammed fibroblasts show global epigenetic remodeling and widespread tissue contribution. Cell Stem Cell *1*, 55–70.

Maxwell, S.L., Ho, H.-Y., Kuehner, E., Zhao, S., and Li, M. (2005). Pitx3 regulates tyrosine hydroxylase expression in the substantia nigra and identifies a subgroup of mesencephalic dopaminergic progenitor neurons during mouse development. Dev. Biol. *282*, 467–479.

Meissner, A., Gnirke, A., Bell, G.W., Ramsahoye, B., Lander, E.S., and Jaenisch, R. (2005). Reduced representation bisulfite sequencing for comparative high-resolution DNA methylation analysis. Nucleic Acids Res. *33*, 5868–5877.

Mellén, M., Ayata, P., Dewell, S., Kriaucionis, S., and Heintz, N. (2012). MeCP2 binds to 5hmC enriched within active genes and accessible chromatin in the nervous system. Cell *151*, 1417–1430.

Mohn, F., Weber, M., Rebhan, M., Roloff, T.C., Richter, J., Stadler, M.B., Bibel, M., and Schübeler, D. (2008). Lineage-specific polycomb targets and de novo DNA methylation define restriction and potential of neuronal progenitors. Mol. Cell *30*, 755–766.

Momčilović, O., Liu, Q., Swistowski, A., Russo-Tait, T., Zhao, Y., Rao, M.S., and Zeng, X. (2014). Genome wide profiling of dopaminergic neurons derived from human embryonic and induced pluripotent stem cells. Stem Cells Dev. *23*, 406–420.

Nishino, K., Toyoda, M., Yamazaki-Inoue, M., Fukawatase, Y., Chikazawa, E., Sakaguchi, H., Akutsu, H., and Umezawa, A. (2011). DNA methylation dynamics in human induced pluripotent stem cells over time. PLoS Genet. *7*, e1002085.

Ohi, Y., Qin, H., Hong, C., Blouin, L., Polo, J.M., Guo, T., Qi, Z., Downey, S.L., Manos, P.D., Rossi, D.J., et al. (2011). Incomplete DNA methylation underlies a transcriptional memory of somatic cells in human iPS cells. Nat. Cell Biol. *13*, 541–549.

Perrier, A.L., Tabar, V., Barberi, T., Rubio, M.E., Bruses, J., Topf, N., Harrison, N.L., and Studer, L. (2004). Derivation of midbrain dopamine neurons from human embryonic stem cells. Proc. Natl. Acad. Sci. USA *101*, 12543–12548.

Polo, J.M., Liu, S., Figueroa, M.E., Kulalert, W., Eminli, S., Tan, K.Y., Apostolou, E., Stadtfeld, M., Li, Y., Shioda, T., et al. (2010). Cell type of origin influences the molecular and functional properties of mouse induced pluripotent stem cells. Nat. Biotechnol. *28*, 848–855.

Ren, Y., Lee, M.Y., Schliffke, S., Paavola, J., Amos, P.J., Ge, X., Ye, M., Zhu, S., Senyei, G., Lum, L., et al. (2011). Small molecule Wnt inhibitors enhance the efficiency of BMP-4-directed cardiac differentiation of human pluripotent stem cells. J. Mol. Cell. Cardiol. *51*, 280–287.

Roessler, R., Boddeke, E., and Copray, S. (2013). Induced pluripotent stem cell technology and direct conversion: new possibilities to study and treat Parkinson's disease. Stem Cell Rev. *9*, 505–513.

Rudenko, A., Dawlaty, M.M., Seo, J., Cheng, A.W., Meng, J., Le, T., Faull, K.F., Jaenisch, R., and Tsai, L.-H. (2013). Tet1 is critical for neuronal activity-regulated gene expression and memory extinction. Neuron *79*, 1109–1122.

Salti, A., Nat, R., Neto, S., Puschban, Z., Wenning, G., and Dechant, G. (2013). Expression of early developmental markers predicts the efficiency of embryonic stem cell differentiation into midbrain dopaminergic neurons. Stem Cells Dev. *22*, 397–411.

Shao, K., Koch, C., Gupta, M.K., Lin, Q., Lenz, M., Laufs, S., Denecke, B., Schmidt, M., Linke, M., Hennies, H.C., et al. (2013). Induced pluripotent mesenchymal stromal cell clones retain donor-derived differences in DNA methylation profiles. Mol. Ther. *21*, 240–250.

Smallwood, S.A., Tomizawa, S.-I., Krueger, F., Ruf, N., Carli, N., Segonds-Pichon, A., Sato, S., Hata, K., Andrews, S.R., and Kelsey, G. (2011). Dynamic CpG island methylation landscape in oocytes and preimplantation embryos. Nat. Genet. *43*, 811–814.

Smidt, M.P., and Burbach, J.P.H. (2007). How to make a mesodiencephalic dopaminergic neuron. Nat. Rev. Neurosci. *8*, 21–32.

Smidt, M.P., Smits, S.M., and Burbach, J.P. (2003). Molecular mechanisms underlying midbrain dopamine neuron development and function. Eur. J. Pharmacol. *480*, 75–88.

Smidt, M.P., Smits, S.M., Bouwmeester, H., Hamers, F.P., van der Linden, A.J., Hellemons, A.J., Graw, J., and Burbach, J.P. (2004). Early developmental failure of substantia nigra dopamine neurons in mice lacking the homeodomain gene Pitx3. Development *131*, 1145–1155.

Sullivan, G.J., Bai, Y., Fletcher, J., and Wilmut, I. (2010). Induced pluripotent stem cells: epigenetic memories and practical implications. Mol. Hum. Reprod. *16*, 880–885.

Sundberg, M., Bogetofte, H., Lawson, T., Jansson, J., Smith, G., Astradsson, A., Moore, M., Osborn, T., Cooper, O., Spealman, R., et al. (2013). Improved cell therapy protocols for Parkinson's disease based on differentiation efficiency and safety of hESC-, hiPSC-, and non-human primate iPSC-derived dopaminergic neurons. Stem Cells *31*, 1548–1562.

Swistowski, A., Peng, J., Liu, Q., Mali, P., Rao, M.S., Cheng, L., and Zeng, X. (2010). Efficient generation of functional dopaminergic neurons from human induced pluripotent stem cells under defined conditions. Stem Cells *28*, 1893–1904.

Takahashi, K., and Yamanaka, S. (2006). Induction of pluripotent stem cells from mouse embryonic and adult fibroblast cultures by defined factors. Cell *126*, 663–676.

van Heesbeen, H.J., Mesman, S., Veenvliet, J.V., and Smidt, M.P. (2013). Epigenetic mechanisms in the development and maintenance of dopaminergic neurons. Development *140*, 1159–1169.

Veenvliet, J.V., Dos Santos, M.T., Kouwenhoven, W.M., von Oerthel, L., Lim, J.L., van der Linden, A.J., Koerkamp, M.J., Holstege, F.C., and Smidt, M.P. (2013). Specification of dopaminergic subsets involves interplay of En1 and Pitx3. Development *140*, 3373–3384.

Watanabe, D., Uchiyama, K., and Hanaoka, K. (2006). Transition of mouse de novo methyltransferases expression from Dnmt3b to Dnmt3a during neural progenitor cell development. Neuroscience *142*, 727–737.

Wernig, M., Zhao, J.-P., Pruszak, J., Hedlund, E., Fu, D., Soldner, F., Broccoli, V., Constantine-Paton, M., Isacson, O., and Jaenisch, R. (2008). Neurons derived from reprogrammed fibroblasts functionally integrate into the fetal brain and improve symptoms of rats with Parkinson's disease. Proc. Natl. Acad. Sci. USA *105*, 5856–5861.

Yamaguchi, M., Saito, H., Suzuki, M., and Mori, K. (2000). Visualization of neurogenesis in the central nervous system using nestin promoter-GFP transgenic mice. Neuroreport *11*, 1991–1996.

Zhang, R.-R., Cui, Q.Y., Murai, K., Lim, Y.C., Smith, Z.D., Jin, S., Ye, P., Rosa, L., Lee, Y.K., Wu, H.-P., et al. (2013). Tet1 regulates adult hippocampal neurogenesis and cognition. Cell Stem Cell *13*, 237–245.

Zhao, S., Maxwell, S., Jimenez-Beristain, A., Vives, J., Kuehner, E., Zhao, J., O'Brien, C., de Felipe, C., Semina, E., and Li, M. (2004). Generation of embryonic stem cells and transgenic mice expressing green fluorescence protein in midbrain dopaminergic neurons. Eur. J. Neurosci. *19*, 1133–1140.

Stem Cell Reports, Volume *2*

# Supplemental Information

# Detailed Analysis of the Genetic and Epigenetic Signatures of iPSC-Derived Mesodiencephalic Dopaminergic Neurons

Reinhard Roessler, Sebastien A. Smallwood, Jesse V. Veenvliet, Petros Pechlivanoglou, Su-Ping Peng, Koushik Chakrabarty, Marian J.A. Groot-Koerkamp, R. Jeroen Pasterkamp, Evelyn Wesseling, Gavin Kelsey, Erik Boddeke, Marten P. Smidt, and Sjef Copray

## Inventory of Supplemental Information

### Supplemental Experimental Procedures:

*FAC sorting*

*Electrophysiology*

*Rat rotation model*

*Immunocytochemistry*

*Western Blot*

*Direct bisulfite sequencing*

*Quantitative PCR (qPCR)*

*Quantitative dopamine determination by LC-MS/MS*

*Reverse transcriptase PCR*

*Gene ontology analysis*

### Supplemental References

### Supplemental Table: 1

**Table S1:** Primer sequences used for RT- and quantitative RT-PCR

**Supplemental Figures: 4**

**Figure S1**, related to Figure 1: Generation of iPSCs from *Pitx3*$^{Gfp/+}$ MEFs.

**Figure S2**, related to Figure 1: PITX3-GFP sorted DA neurons function *in vivo*

**Figure S3**, related to Figure 2: Extended expression profiling of mdDA signature.

**Figure S4**, related to Figure 3: DNA methylation profile for key DA transcription factors.

**Figure S5**, related to Figure 4: Analysis of gene expression profiles vs. DNA methylation patterns in mdDa neurons and iPSC-DA neurons respectively.

## Supplemental Experimental Procedures

*FAC sorting*

Freshly dissected PITX3-GFP positive ventral midbrains from several developmental stages, undifferentiated and differentiated iPSC-derived cells were dissociated using the Papain dissociation Kit (Worthington Biochem, Products, LK003150). Dissociated cells were collected in colourless DMEM (Gibco) with 20 ng/ml BDNF, 20 ng/ml GDNF, 0.2 mM ascorbic acid, 1 ng/ml TGFb3 and 0.1 mM dbcAMP, and sorted on a MoFlo-XDP or MoFlo-Astrios sorter with a 100 um nozzle at a pressure of 15-17 psi based on GFP expression. Cells were collected in N2 medium with BDNF, ascorbic acid, GDNF, TGFb3 and dbcAMP or cells were collected in PBS, pelleted and used for RNA and DNA isolation.

*Electrophysiology*

For patch clamp experiments, FAC-sorted iPSC-derived *Pitx3*$^{Gfp/+}$ cells were spun down at 800 rcf for 10 min, resuspended and plated on mouse astrocyte feeders. Electrophysiological recordings were performed on *Pitx3*$^{Gfp/+}$ neurons one week after plating. A standard whole-cell patch clamp technique was used to measure currents at room temperature (22–24°C) using Axopatch 200B amplifier, Digidata 1320A, and pClamp version 8.2 software (Axon Instruments, Foster City, CA). Extracellular bath solution contained (in mmol/L): 140 NaCl, 4 KCl, 5 glucose, 2 $CaCl_2$, 1 $MgCl_2$, and 10 HEPES, pH adjusted to 7.4 with NaOH. Pipette solution contained (in mmol/L) 140 KCl, 10 EGTA, 1 $CaCl_2$, 1 $MgCl_2$ and 10 HEPES,2 $Na_2ATP$, pH adjusted to 7.2 with KOH and had an osmolality of 290-300 mOsm. Microelectrodes were made of GC120F- 10 borosilicate glass (Harvard Apparatus) and pulled on a P-87 puller (Sutter Instruments, Novato, CA) having a final resistance of 4–5 MΩ. Series resistance was compensated by 80%–85%. Currents were filtered at 5 kHz and digitized at 10 kHz. Data were analyzed using Clampfit (Axon Instruments) and Excel (Microsoft). In the current clamp mode cell membrane potential was held at −50 mV by injecting a steady holding current. Cells were activated with a 500 ms pulse of depolarizing current.

*Rat rotation model*

To test functionality of iPSC-derived DA neurons, we have used the well-established rat rotation model (Ungerstedt, 1971). Adult female Sprague Dawley (Harlan) rats (180–230g) were housed under standard conditions with free access to food and water. All animal experiments were carried out

according to the Dutch Regulations for Animal Welfare. Protocols were approved by the Institutional Animal Care and Use Committee of the University of Groningen. Rats were anaesthetized with ketamine (90mg/kg) and xylazine (4mg/kg). Unilateral retrograde destruction of dopaminergic neurons in the substantia nigra was induced by 2 stereotaxic injections of 2.5 µl 6-OHDA (3 mg/ml in 0.2% ascorbic acid and 0.9% saline, Sigma) in the medial forebrain bundle of the nigro-striatal pathway (stereotaxic coordinates 1: AP -4.0, ML −0.8 and V −8.0; tooth bar set at +3.4. and coordinates 2: AP -4.4, ML −1.2 and V −7.8; tooth bar set at −2.4). Unilateral destruction of the nigro-striatal pathway was established by recording of d-amphetamine (5mg/kg)-induced rotation behavior after 3, 6 and 9 weeks. Rats were selected for transplantation if the number of rotations exceeded 4 per min at 6–8 weeks post injection. Under ketamine/metedomedine anesthesia, 2-3*$10^4$ cells were stereotactically transplanted into the striatum (coordinates: AP +0.9mm, ML −2.6mm and V −4.0mm; tooth bar set at 0.0); control rats received a similar injection with mouse embryonic fibroblasts. Daily intraperitoneal injections of cyclosporine 15mg/kg (UMCG pharmacy) were given, starting 24 h before cell grafting (double dosage), and continued until the rats were sacrificed and perfusion fixated at 9 weeks after grafting. Under ketamine/metedomedine anesthesia, rats were transcardially perfused with 4% paraformaldehyde (PFA) in PBS. Brains were explanted, post-fixed in 4% PFA for 24 hours and soaked in a 20 % sucrose solution for 1 day. Brains were sectioned (14µm sections) on a cryostat after embedding in O.C.T. compound (Sakura Finetek, Torrance, USA).

*Immunocytochemistry*

Cells were fixed in 4% paraformaldehyde for 15 min and blocked with 5% normal goat serum, 2% fetal calf serum in 0,1% Triton X in PBS. To characterize iPSC colonies, primary antibodies for Nanog (1:500, Abcam, ab80892), Oct3/4 (1:500, sc-5279, Santa Cruz), Klf4 (1:500, Abcam, ab72543), Sox2 (1:500, Abcam, ab15830), UTF1 (1:1000, mUTF1 custom made rabbit polyclonal, Eurogentec, (Van den Boom et al., 2007)) and alkaline phosphatase (1:500, Abcam, ab65834) were used. To characterize iPSC-derived neurons, primary antibodies for Map2 (1:500, Millipore, AB5622), TH (1:1000, Millipore, ab152) and GFP (1:500, Abcam, ab290) were used. To visualize primary antibodies, fluorescently labeled secondary anti-mouse and anti-rabbit antibodies were applied. Nuclear staining was performed using Hoechst.

*Western Blot*

SDS-PAGE gel electrophoresis and Western Blot analysis were performed to detect Nanog, Oct3/4, Sox2 and UTF1. The following antibodies were used: rabbit anti-Nanog antibody (1:1000, ab80892, Abcam, Cambridge, UK), mouse Oct3/4 antibody (1:500, sc-5279, Santa Cruz), rabbit anti-Sox2 (1:4000, ab15830, Abcam, Cambridge, UK), rabbit UTF1 (1:2000) and mouse anti-β-Actin (ab6276, Abcam, Cambridge, UK) at 1:10000 dilution. The housekeeping gene β-Actin has been used as loading control. Primary antibodies were detected using fluorescently labeled secondary antibody: donkey anti-mouse (IRDye® 680, LI-COR, Biosciences) and donkey anti-rabbit (IRDye® 800CW, LI-COR, Biosciences) according to manufactures instructions.

*Direct bisulfite sequencing*

Genomic DNA from Pitx3$^{gfp/+}$ iPSCs was purified by proteinase K digestion followed by phenol-chloroform extraction. Sodium bisulfite conversion was carried out with the Zymo EZ DNA methylation Kit. PCRs were performed for Oct4 (FW: 5'-GTGTAGTGTTAATAGGTTTT-GTGG-3'; BW: 5' TATTTTTTAATTTTAA-TTTTTA3'), Nanog (FW: 5'GGATTTTGTAGG-TGGGATTAAT 3'; BW: 5' TAAACAACAACCAAAAAACTCAA 3'). Sequence analysis of converted fragments was done using Quma Quantification tool for methylation analysis (quma.cdb.riken.jp).

*Quantitative PCR (qPCR)*

Relative gene expression levels were determined by qPCR real-time pcr (Lightcycler) using the QuantiTect™ SYBR® Green PCR (QIAGEN) LightCycler® kit (Roche, Idaho Technologies) according to the manufacturer's instructions. For each reaction 0.1ng total RNA from FAC-sorted neurons was used as input. Relative expression levels were normalized to housekeeping gene *18s*. Primer sequences see Table 1.

*Quantitative dopamine determination by LC-MS/MS*

Dopamine production was determined as described earlier (Van de Merbel et al., 2011). Instead of plasma we analyzed 100µl of supernatant or cell homogenate from terminally DA differentiated iPSCs or undifferentiated iPSCs (3 technical replicates each) for the derivation step.

*Reverse transcriptase PCR*

Briefly, RNA was isolated using the RNA-easy kit (Qiagen). To characterize endogenous expression of pluripotency markers in *Pitx3*$^{Gfp/+}$ iPSC clones 3 and 5, the following primer genes were analyzed: Nanog, Oct4, Sox2, Klf4 and cMyc (primer sequences as published by Takahashi and Yamanaka, 2006, see Table 1). Expression levels were compared between two iPSC clones, ES cell line IB10 and mouse embryonic fibroblasts (C57BL/6).

*Gene ontology analysis*

GO analysis on transcriptome data was performed using the BiNGO 2.44 plug-in (Cytoscape 2.8.2; Hypergeometric test; FDR correction) using genes with an at least 2-fold up– or down-regulation in iPSC-derived DA neurons as compared to their primary counterpart at any developmental time point as input, and all annotated genes on the micro-array as reference. GO analysis for the RRBS data set (see below for RRBS) was performed using online functional annotation tool DAVID (Huang et al., 2009). The list of genes analyzed with DAVID was generated based on the entire RRBS data set. To select most different genes CGI methylation reads were sorted and plotted after logit transformation (see Figure S5). Histogram plots were created with the statistical software R v.2.15.2 [R Core Team (2012).

## Supplemental References


Hargus, G., Cooper, O., Deleidi, M., Levy, A., Lee, K., Marlow, E., Yow, A., Soldner, F., Hockemeyer, D., Hallett, P.J., Osborn, T., Jaenisch, R., Isacson, O. (2010). Differentiated Parkinson patient-derived induced pluripotent stem cells grow in the adult rodent brain and reduce motor asymmetry in Parkinsonian rats. Proc. Natl. Acad. Sci. USA *107*, 15921–6.

Ungerstedt, U. (1971). Postsynaptic supersensitivity after 6-hydroxy-dopamine induced degeneration of the nigro-striatal dopamine system. Acta Physiol. Scand. Suppl. *367*, 69–93.

Van de Merbel, N.C., Hendriks, G., Imbos, R., Tuunainen, J., Rouru, J., Nikkanen, H. (2011). Quantitative determination of free and total dopamine in human plasma by LC-MS/MS: the importance of sample preparation. Bioanalysis *3*, 1949–61.

Van den Boom, V., Kooistra, S.M., Boesjes, M., Geverts, B., Houtsmuller, A.B., Monzen, K., Komuro, I., Essers, J., Drenth-Diephuis, L.J., Eggen, B. (2007). UTF1 is a chromatin-associated protein involved in ES cell differentiation. J. Cell Biol. *178*, 913–24.

**Table S1: Primer sequences used for RT- and quantitative RT-PCR**

| Gene | Forward primer (5' to 3') | Reverse primer (5' to 3') |
|---|---|---|
| ***RT-PCR*** | | |
| *Nanog* | CAGGTGTTTGAGGGTAGCTC | CGGTTCATCATGGTACAGTC |
| *Oct4* | TCTTTCCACCAGGCCCCCGGCTC | TGCGGGCGGACATGGGGAGATCC |
| *Sox2* | TAGAGCTAGACTCCGGGCGATGA | TTGCCTTAAACAAGACCACGAAA |
| *cMyc* | TGACCTAACTCGAGGAGGAGCTGG AATC | AAGTTTGAGGCAGTTAAAATTATGG CTGAAGC |
| *Klf4* | GCGAACTCACACAGGCGAGAAACC | TCGCTTCCTCTTCCTCCGACACA |
| *GAPDH* | CATCAAGAAGGTGGTGAAGC | ACCACCCTGTTGCTGTAG |
| ***qRT*** | | |
| *18s* | AAACGGCTACCACATCCAAG | CCTCCAATGGATCCTCGTTA |
| *Desmin* | GCGGCTAAGAACATCTCTGAGG | ATCTCGCAGGTGTAGGACTGGA |
| *Dlk1* | TGGCTGTGTCAATGGAGTCTGC | CCACGCAAGTTCCATTGTTGGC |
| *En1* | GGTCTACTGCACACGCTATTCG | AACTCCGCCTTGAGTCTCTGCA |
| *En2* | GGTCTACTGCACGCGCTATTCT | AAACTCAGCCTTGAGCCTCTGG |
| *Nurr1* | CCGCCGAAATCGTTGTCAGTAC | TTCGGCTTCGAGGGTAAACGAC |
| *Pitx3* | CTTCCAGAGGAATCGCTACCCT | CTGCGAAGCCACCTTTGCACAG |
| *Sox9* | CACACGTCAAGCGACCCATGAA | TCTTCTCGCTCTCGTTCAGCAG |

## Figure S1, related to Figure 1

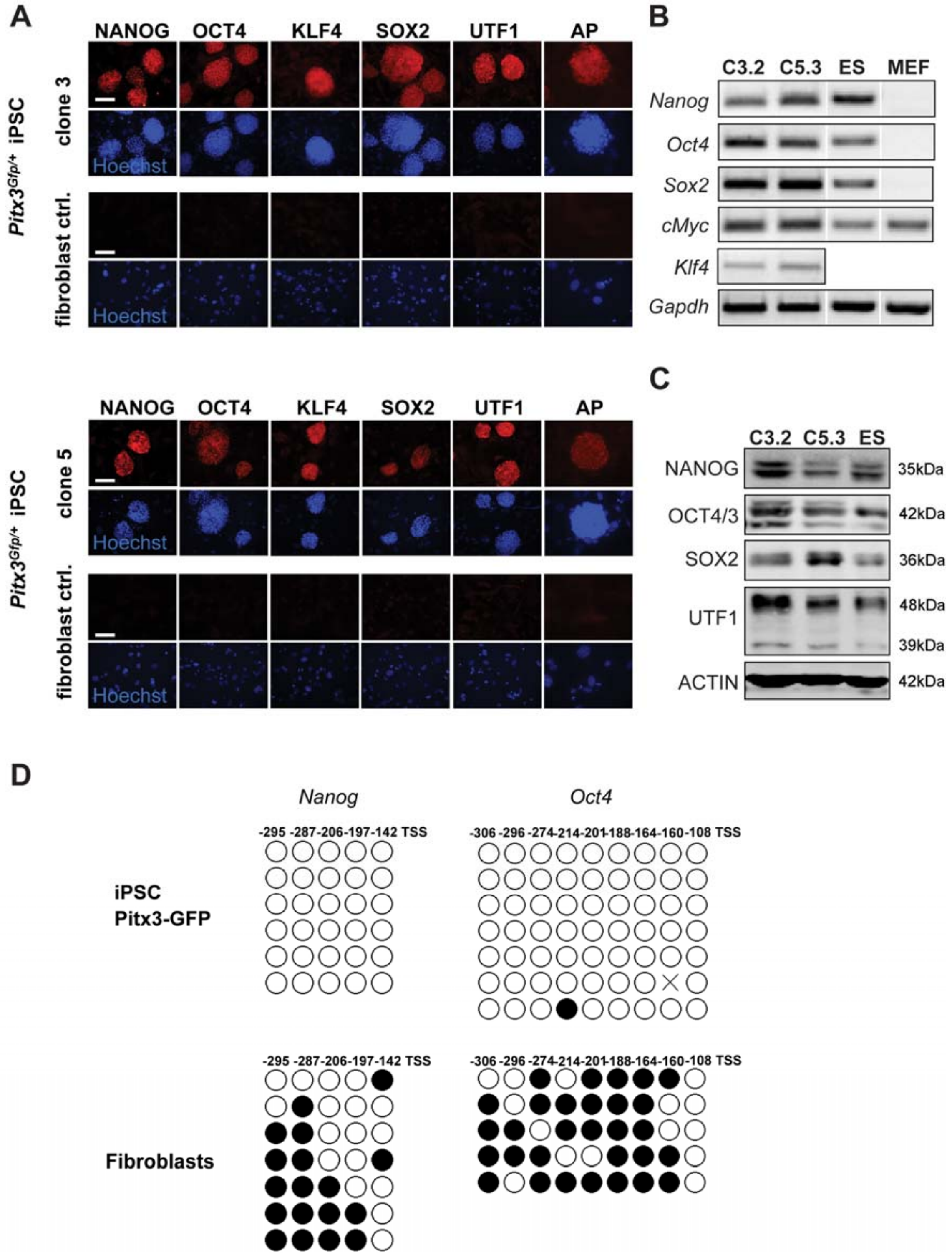


**Figure S1, related to Figure 1: Generation of iPSCs from *Pitx3$^{Gfp/+}$* MEFs. (A)** Characterization of iPSC clone 3 and 5 using immune reactivity for selected pluripotency markers (blue=Hoechst nuclear staining; red=immunostaining for the indicated (transcription) factor) Scale bars for all: 100µm. **(B)** Endogenous expression of pluripotency markers comparing iPSC clones 3 and 5 with embryonic stem cells (ES) and MEFs. **(C)** Protein expression of selected pluripotency markers comparing iPSC clones

3 and 5 with ES cells. **(D)** Bisulfite sequencing of promoter regions of Nanog and Oct4 comparing *Pitx3*$^{Gfp/+}$ iPSCs with *Pitx3*$^{Gfp/+}$ MEFs. Open circles represent unmethylated CpG dinucleotides, whereas closed circles indicate methylated CpG dinucleotides.

## Figure S2, related to Figure 1

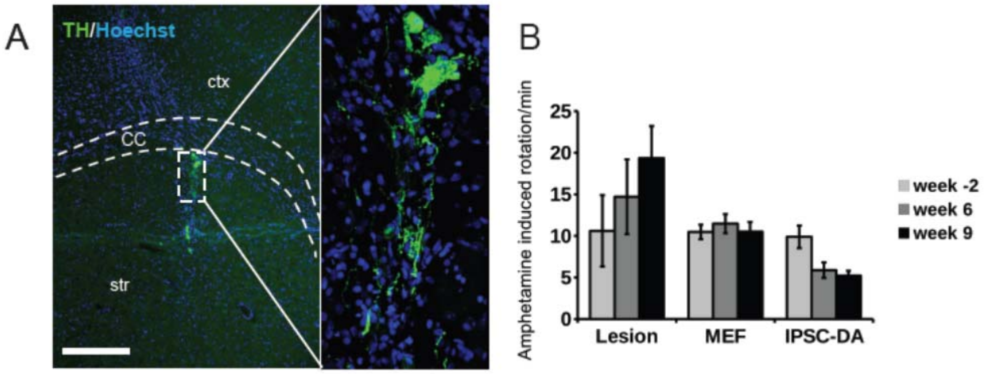


**Figure S2, related to Figure 1: PITX3-GFP sorted DA neurons function *in vivo*** (A) Immunohistochemistry of a section of the upper part of the rat striatum showing some of the grafted TH-positive iPSC-derived mdDA neurons with their extensive outgrowth. Scale bar = 1mm; CC=corpus callosum; str=striatum; ctx=cortex. (B) Amphetamine-induced rotation in purified *Pitx3*$^{Gfp/+}$ neuron grafted animals (n=7 ) vs. controls (only lesioned, n=2 and grafted MEFs, n=3; error bar = standard deviation around mean).

## Figure S3, related to Figure 2

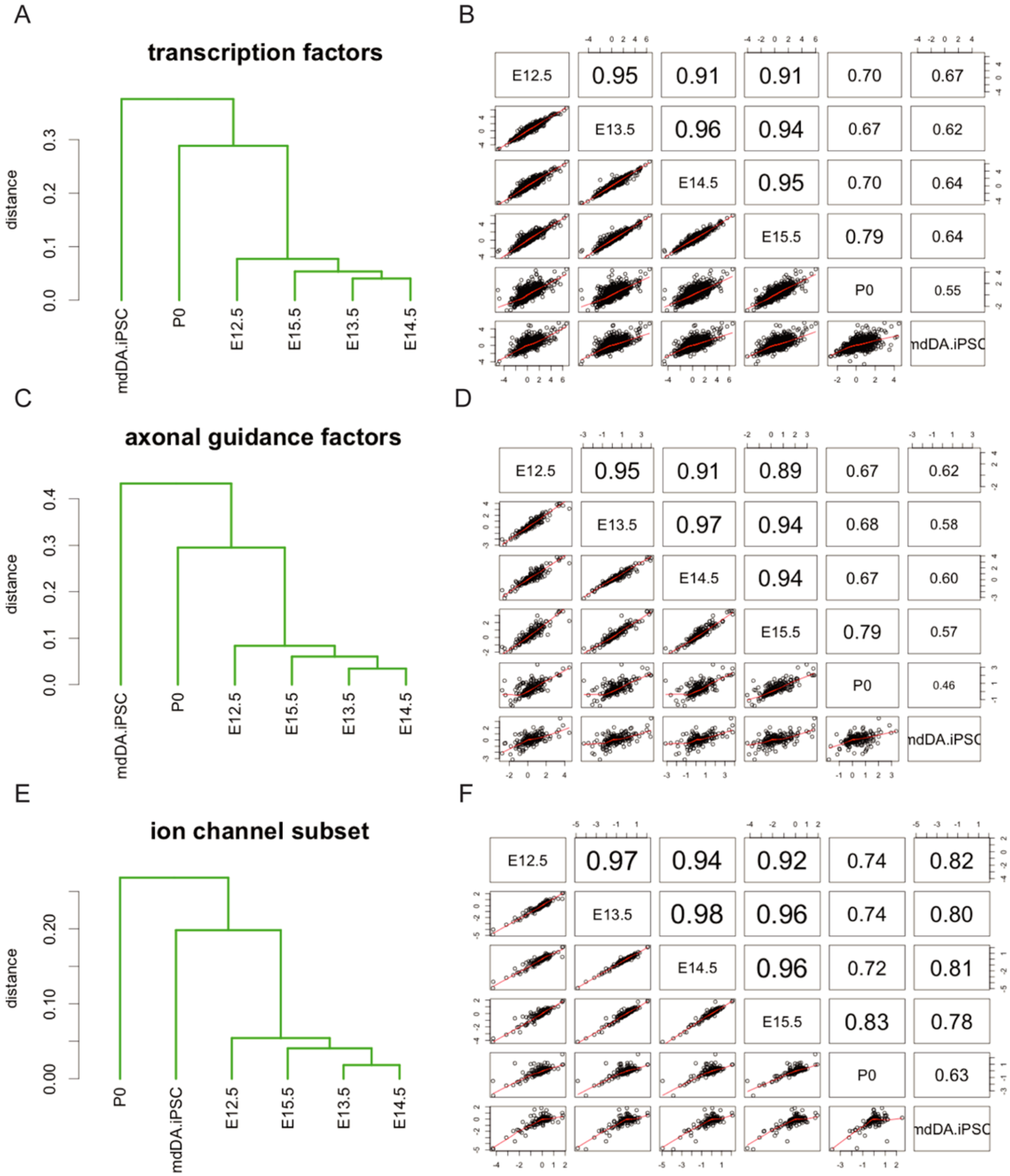


**Figure S3, related to Figure 2: Extended expression profiling of mdDA signature.** (A) Hierarchical clustering of DA-specific transcription factors. (B) Comprehensive analysis of expression correlation for DA-specific transcription factors. (C) Hierarchical clustering of axonal guidance factors. (D) Comprehensive analysis of expression correlation for axonal guidance factors. (E) Hierarchical clustering of ion channel subset. (F) Comprehensive analysis of expression correlation for ion channel subset.

## Figure S4, related to Figure 3

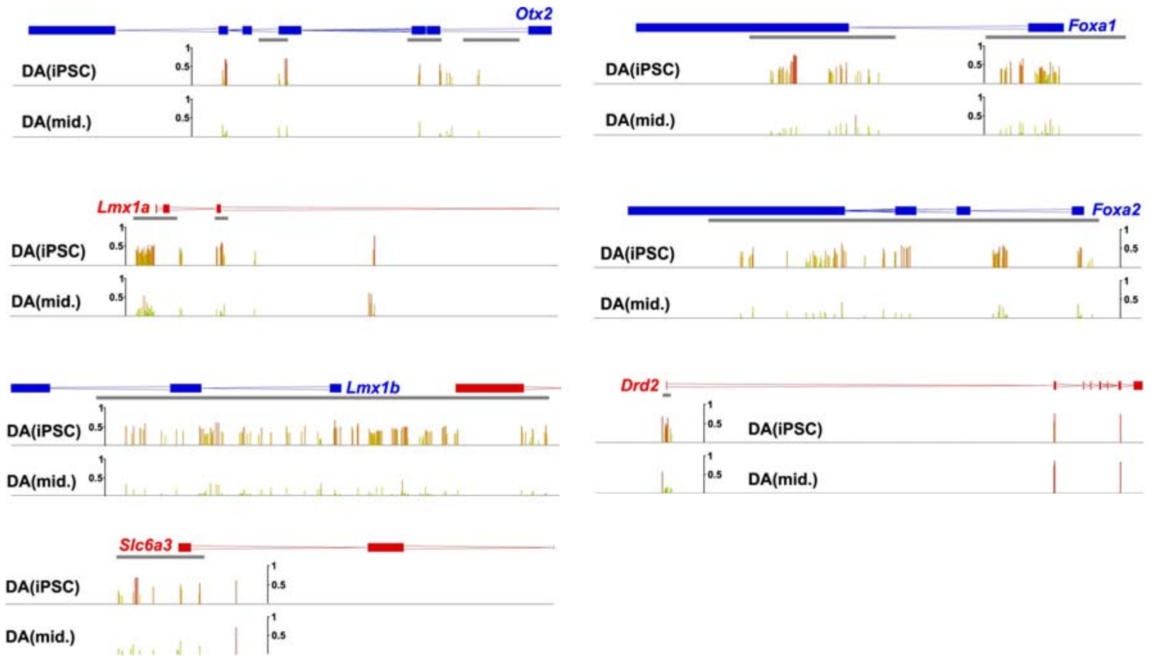


**Figure S4, related to Figure 3: DNA methylation profile for key DA transcription factors.** DNA methylation profile of the complete gene bodies highlighting identified CGIs for *Otx2, Lmx1a, Lmx1b*, *FoxA1/2, Slc6a3* (aka Vmat2) and *Drd2*. Most linage-specific differences can be seen in putative promoter areas. Color and arrows provide information regarding gene orientation (red = + strand; blue = - strand). Methylation across GCIs is represented in ‰.

## Figure S5, related to Figure 4

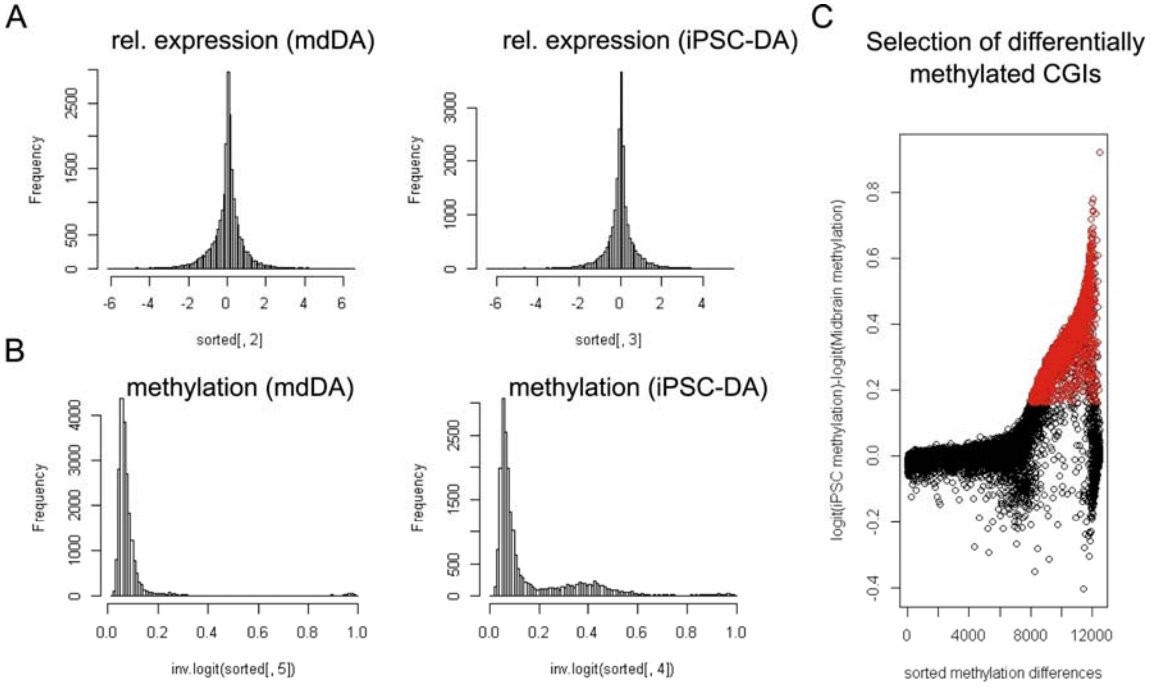


**Figure S5, related to Figure 4: Analysis of gene expression profiles vs. DNA methylation patterns in mdDA neurons and iPSC-DA neurons respectively. (A)** Relative global expression levels in mdDA- and iPSC-DA neurons. **(B)** Distribution of global DNA Methylation in mdDA and iPSC-DA neurons. **(C)** Identification and selection of differentially methylated CGIs in iPSC-DA neurons compared to mdDA neurons. CGIs labeled in red were selected for GO term analysis (see Figure 5).
